# A protein complex required for polar growth of rhizobial infection threads

**DOI:** 10.1038/s41467-019-10029-y

**Published:** 2019-06-28

**Authors:** Cheng-Wu Liu, Andrew Breakspear, Nicola Stacey, Kim Findlay, Jin Nakashima, Karunakaran Ramakrishnan, Miaoxia Liu, Fang Xie, Gabriella Endre, Fernanda de Carvalho-Niebel, Giles E. D. Oldroyd, Michael K. Udvardi, Joëlle Fournier, Jeremy D. Murray

**Affiliations:** 1 Cell and Developmental Biology, John Innes Centre, Norwich Research Park, Norwich, NR4 7UH UK; 2 Noble Research Institute, 2510 Sam Noble Parkway, Ardmore, OK 73401 USA; 3 National Key Laboratory of Plant Molecular Genetics, CAS Center for Excellence in Molecular and Plant Sciences, Institute of Plant Physiology and Ecology, Chinese Academy of Sciences, 200032 Shanghai, China; 4 Institute of Plant Biology, Biological Research Centre, Szeged, 6726 Hungary; 5 LIPM, Université de Toulouse, INRA, CNRS, 31326 Castanet-Tolosan, France; 6 Sainsbury Laboratory, University of Cambridge, 47 Bateman Street, Cambridge, CB2 1LR UK; 7 National Key Laboratory of Plant Molecular Genetics, CAS-JIC Centre of Excellence for Plant and Microbial Science (CEPAMS), CAS Center for Excellence in Molecular and Plant Sciences, Institute of Plant Physiology and Ecology, Chinese Academy of Sciences, Shanghai, 200032 China; 8 Present Address: Sainsbury Laboratory, University of Cambridge, 47 Bateman Street, Cambridge, CB2 1LR UK

**Keywords:** Exocytosis, Plant cell biology, Rhizobial symbiosis

## Abstract

During root nodule symbiosis, intracellular accommodation of rhizobia by legumes is a prerequisite for nitrogen fixation. For many legumes, rhizobial colonization initiates in root hairs through transcellular infection threads. In *Medicago truncatula*, VAPYRIN (VPY) and a putative E3 ligase LUMPY INFECTIONS (LIN) are required for infection thread development but their cellular and molecular roles are obscure. Here we show that LIN and its homolog LIN-LIKE interact with VPY and VPY-LIKE in a subcellular complex localized to puncta both at the tip of the growing infection thread and at the nuclear periphery in root hairs and that the punctate accumulation of VPY is positively regulated by LIN. We also show that an otherwise nuclear and cytoplasmic exocyst subunit, EXO70H4, systematically co-localizes with VPY and LIN during rhizobial infection. Genetic analysis shows that defective rhizobial infection in *exo70h4* is similar to that in *vpy* and *lin*. Our results indicate that VPY, LIN and EXO70H4 are part of the symbiosis-specific machinery required for polar growth of infection threads.

## Abstract (web summary)

Many legumes accommodate rhizobial symbionts via transcellular infection threads. Here the authors show that in *Medicago* root hairs, polar growth of the infection thread requires a tip-localized protein complex consisting of VPY and VPY-like proteins that are stabilized by the E3 ligase LIN, as well as an exocyst complex subunit.

## Introduction

Legumes form nitrogen-fixing symbiosis with soil bacteria called rhizobia, which reduce atmospheric nitrogen to ammonia in the nodule, a specialised plant organ developed from pericycle and cortical tissues of the root^1,2^. For nitrogen fixation to occur it is essential for rhizobia from the rhizosphere to colonize epidermal and outer cortical cells before being taken up into cells of the developing nodule^3^. This colonization process is called infection and is finely coordinated with nodule development^4,5^. In most legumes, including the model species *Medicago truncatula* and *Lotus japonicus*, rhizobial infection initiates in root hairs after perception of rhizobia-produced signalling molecules known as Nodulation (Nod) factors by specific receptors of the host legume^6,7^. Downstream of these Nod factor receptors are various cellular components required for nuclear-associated calcium signalling, involving the generation and decoding of sustained intranuclear calcium spiking^8–12^. This so-called common symbiosis signalling pathway is shared by the arbuscular mycorrhizal symbiosis and is likely to also operate in the *Frankia*-actinorhizal symbiosis^13,14^. During nodulation, activation of the common symbiosis signalling pathway triggers a transcriptional cascade which results in the cellular reprogramming required for intracellular rhizobial infection^15–26^.

Rhizobial entry takes place via the infection thread, a transcellular tubular structure formed by targeted secretion of plant cell wall material and plasma membrane invagination in advance of rhizobial colonization^27–29^. Infection thread formation involves several sequential steps. Rhizobial attachment to the tip of a growing root hair leads to tip-growth reorientation, entrapment of the bacteria between root hair cell walls and the formation of a closed infection chamber^29^. A rhizobial microcolony is formed within the radially expanding infection chamber, from which the tubular infection thread initiates. The infection thread progressively elongates via polar tip growth, following the path of the migrating nucleus along and within the root hair cell. Throughout this process, the nucleus remains in close proximity, connected initially to the infection chamber and then to the growing tip of the infection thread by an ER-rich cytoplasmic bridge^27,30^. This process is repeated in underlying root cortical cell layers until the infection threads ramify within the cells of the developing nodule. The rhizobia are then released intracellularly from the infection threads to form symbiosomes where they differentiate into nitrogen-fixing bacteroids^31^.

The development of the infection chamber and the infection thread involves extensive membrane expansion and cell wall remodelling which require targeted vesicle fusion and delivery of cargo to the extracellular space. One example is the ENOD11 protein from *M. truncatula*, a proline-rich cell wall protein that is secreted into the infection chamber^29^. Another example is the cell wall modifying enzyme, Nodule Pectate Lyase (NPL), which is required for cell wall remodelling at early stages of rhizobial infection in both *L. japonicus* and *M. truncatula*, and is delivered to the infection chamber and infection thread wall^32,33^. The delivery of materials necessary for infection chamber and thread development is thought to involve the host exocytosis machinery including Vesicle-Associated Membrane Proteins such as VAMP721d/e^30,32^. However, the molecular and cellular mechanisms leading to targeted exocytosis and the switch from infection chamber expansion to polar growth of the infection thread in root hairs remain to be established.

Genetic analyses in both *M. truncatula* and *L. japonicus* have identified several of the host components that regulate progression of the infection thread following initiation. These include a coiled-coil protein Required for Polar Growth (RPG)^34^, several proteins involved in actin rearrangement^35–38^, an infection thread localized Cystathionine-β-Synthase-like 1 (CBS1)^39^, a putative E3 ligase LUMPY INFECTIONS (LIN)/CERBERUS and a protein of unknown function called VAPYRIN (VPY)^40–42^. Amongst several transcription factors required for infection^15–26^, Nodule Inception (NIN) is necessary for the expression of several infection-related genes including *NPL*, *RPG* and *CBS1*^32,33,39^. Furthermore, infection chamber development is defective in the *nin* mutant, due to the lack of targeted exocytosis revealed by the absence of VAMP721e accumulation in the surrounding membrane domain^29^.

*vpy* mutants, defective in rhizobial infection, are characterized by underdeveloped small-sized nodules. While the infection chamber and rhizobial microcolony can form in *vpy*, the initiation of infection threads is either delayed or fails to occur^42^. VPY contains an N-terminal Major Sperm protein (MSP) domain and a series of ankyrin repeats at its C-terminus. Since both domains are predicted to mediate protein-protein interactions it is thought that VPY exerts its function by recruiting other protein partners^42–45^. *VPY* is also required for development of arbuscules during mycorrhization and during this process VPY interacts with and partially colocalizes with EXO70I, a subunit of the exocyst complex. The *exo70i* mutant has defective arbuscule development but lacks a nodulation phenotype^46^. No interacting proteins for VPY in nodulation have been reported to date. Here we describe a multi-protein complex comprising VPY, LIN and a component of the exocyst that localizes at the tips of growing infection threads and to a limited number of sites in the cytoplasm. We propose that this infection-associated complex has a central role in polarized growth of the infection thread.

## Results

### *VPY* is required for infection thread initiation and growth

**Fig. 1:**
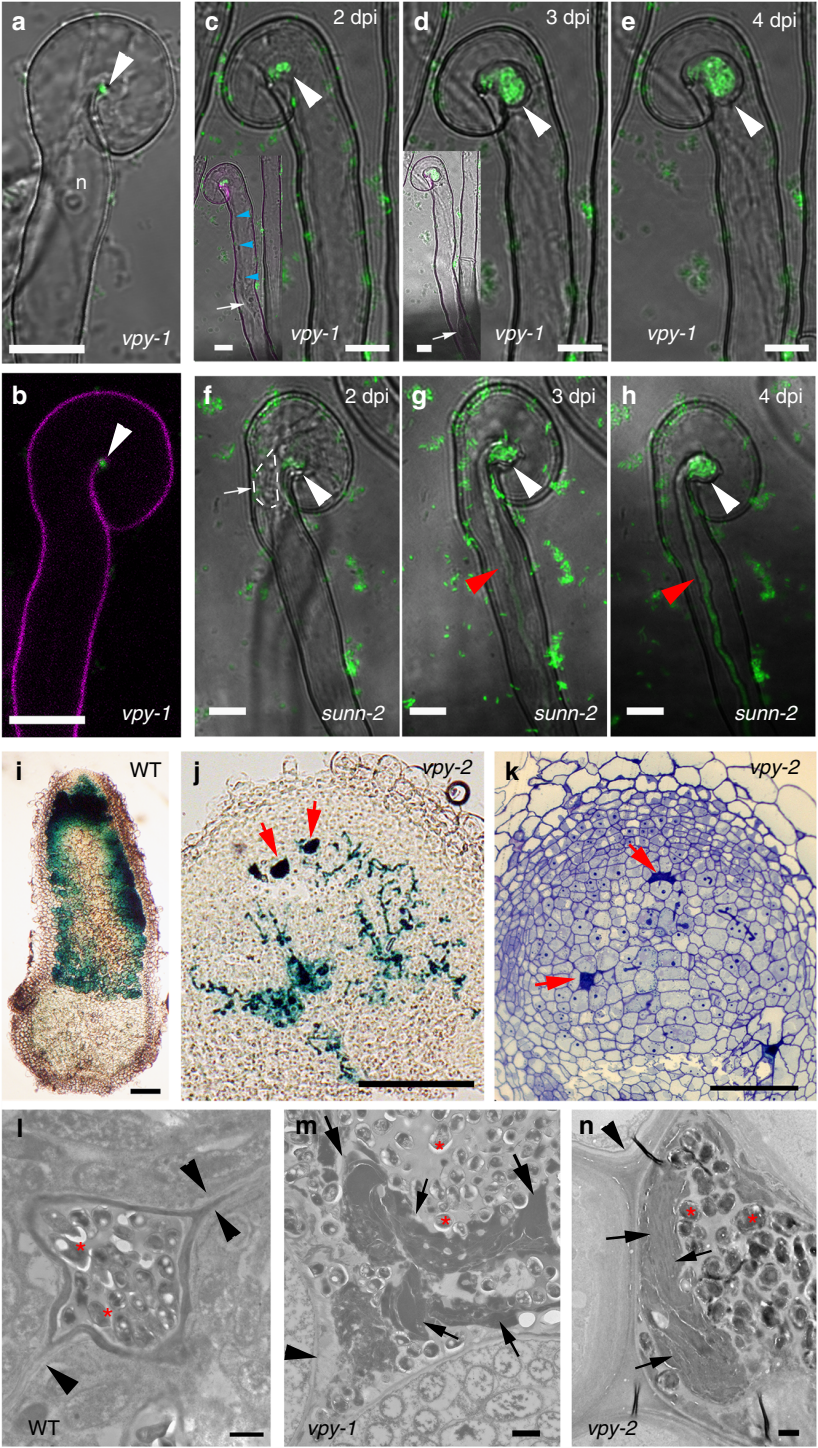
*VPY* is required for rhizobial infection in the epidermal and nodule cells. **a**–**b** Confocal images of *vapyrin-1* (*vpy-1*) mutant showing normal root hair curling and entrapment of rhizobia. Upper, merged image of bright field and GFP; lower, merged pictures of cell wall auto-fluorescence and GFP. **c**–**h** Root hair infection phenotypes of *vpy-1* (**c**–**e**) and control plants (*sunn-2*) (**f**–**h**) at 2, 3 and 4 days post-inoculation (dpi) with Sm2011-GFP, showing exaggerated radial development of the infection chamber and abnormal nucleus positioning in the mutant. White arrows indicate nucleus in **c**, **d** and **f**. Blue arrowheads indicate the cytoplasmic bridge in *vpy-1* (inset in **c**). White arrowheads indicate rhizobia in the infection chamber (green). Red arrowheads indicate elongated infection threads in control plants (**g**, **h**). Insets in **c**, **d** correspond to the same root hair at a lower magnification, showing the positions of nucleus. n, nucleus in **a**. White dashed line indicates the contour of the nucleus in **f**. **i**, **j** Longitudinal nodule sections of X-gal stained WT (**i**) and *vpy-2* (**j**) nodules. **k** Toluidine blue-stained longitudinal nodule section of *vpy-2*. Nodules were collected at 47 dpi. Blue colour **i**, **j** indicates X-gal staining of Rm1021-lacZ. Red arrows indicate abnormal intercellular accumulation of bacteria (**j**, **k**). **l**–**n** Transmission electron microscopy of nodule cells from WT (**l**), *vpy-1* (**m**) and *vpy-2* (**n**) at 47 dpi showing intercellular rhizobial infection. Red asterisks indicate rhizobia. Arrowheads indicate plant cell wall. Arrows indicate unidentified electron dense material accumulating in the intercellular space occupied by rhizobia (**l**–**n**). Scale bars, 10 μm (**a**–**h**), 100 µm (**i**–**k**) and 1 μm (**l**–**n**)

Previous work showed that VPY is required for rhizobial infection of root hairs^42^. To better define how *VPY* contributes to infection thread development in root hairs, we used live cell imaging to compare the earliest stages of root hair infection in the *M. truncatula vpy-1* mutant and in *sunn-2* plants after inoculation with the GFP-tagged *Sinorhizobium meliloti* strain Sm2011 (Sm2011-CFP) (Fig. 1a–h). Supernodulant *sunn-2* plants have a much higher number of infections compared to A17 but are nonetheless wild type-like for infection thread formation which is why they are preferred for in vivo microscopy^30^. Root hair curling and entrapment of rhizobia were normal in *vpy-1* (Fig. 1a, b). However, after the formation of a microcolony within the infection chamber, infection threads did not form in the mutant up to 4 days post-inoculation (dpi) with *S. meliloti* (Fig. 1c-e) in contrast to the wild type situation where infection threads already extended down the root hairs by 3 dpi (Fig. 1f-h). Instead, only swollen infection chambers hosting large microcolonies were found in the mutant 3–4 dpi (Fig. 1c-e), suggesting that, in the absence of VPY, the ability to switch from radial to polar growth of the infection compartment has been lost. In addition, although the nucleus in *vpy-1* curled root hairs was still connected to the developing infection chamber by a cytoplasmic strand, it was no longer in close vicinity of the enclosed rhizobia (Fig. 1c, d insets), in contrast to its usual position adjacent to the developing infection chamber in *sunn-2* root hairs^29^ (Fig. 1f). These results indicate that VPY is also essential for positioning the nucleus close to the developing infection chamber.

Although most rhizobial infections are blocked in root hairs in *vpy-2*, small aberrant nodules do form in mutant plants^47^. We therefore investigated to what extent rhizobial infection was affected in these small nodules. Compared with wild type nodules at the same time point (47 dpi), the nodules of *vpy-2* were much smaller and never elongated (Fig. 1i–k). Within *vpy* nodules, infection threads rarely entered cells and rhizobia frequently accumulated in large intercellular deposits (Fig. 1j, k). Transmission electron microscopy of *vpy-1* and *vpy-2* nodule sections confirmed that these deposits contained large numbers of bacteria but also revealed large amounts of electron dense material (Fig. 1m,  n) that was never observed in wild type nodules (Fig. 1l). This suggests that rhizobial infection thread construction is abnormal in *vpy* nodules, and that secretion processes are altered in the *vpy* mutant. Finally, very few symbiosomes were observed in nodules of *vpy-1* and *vpy-2* (Fig. 1k, Supplementary Fig. 1), most likely as a consequence of defective cortical infection.

Altogether, our data indicate a clear role for VPY in infection thread formation in both root hairs and nodules, and that this role may involve the focused secretion of material that is required for the polar growth of infection threads. We next investigated the spatiotemporal pattern of *VPY* expression and VPY subcellular localization.

### *VPY* expression is associated with rhizobial infection

**Fig. 2:**
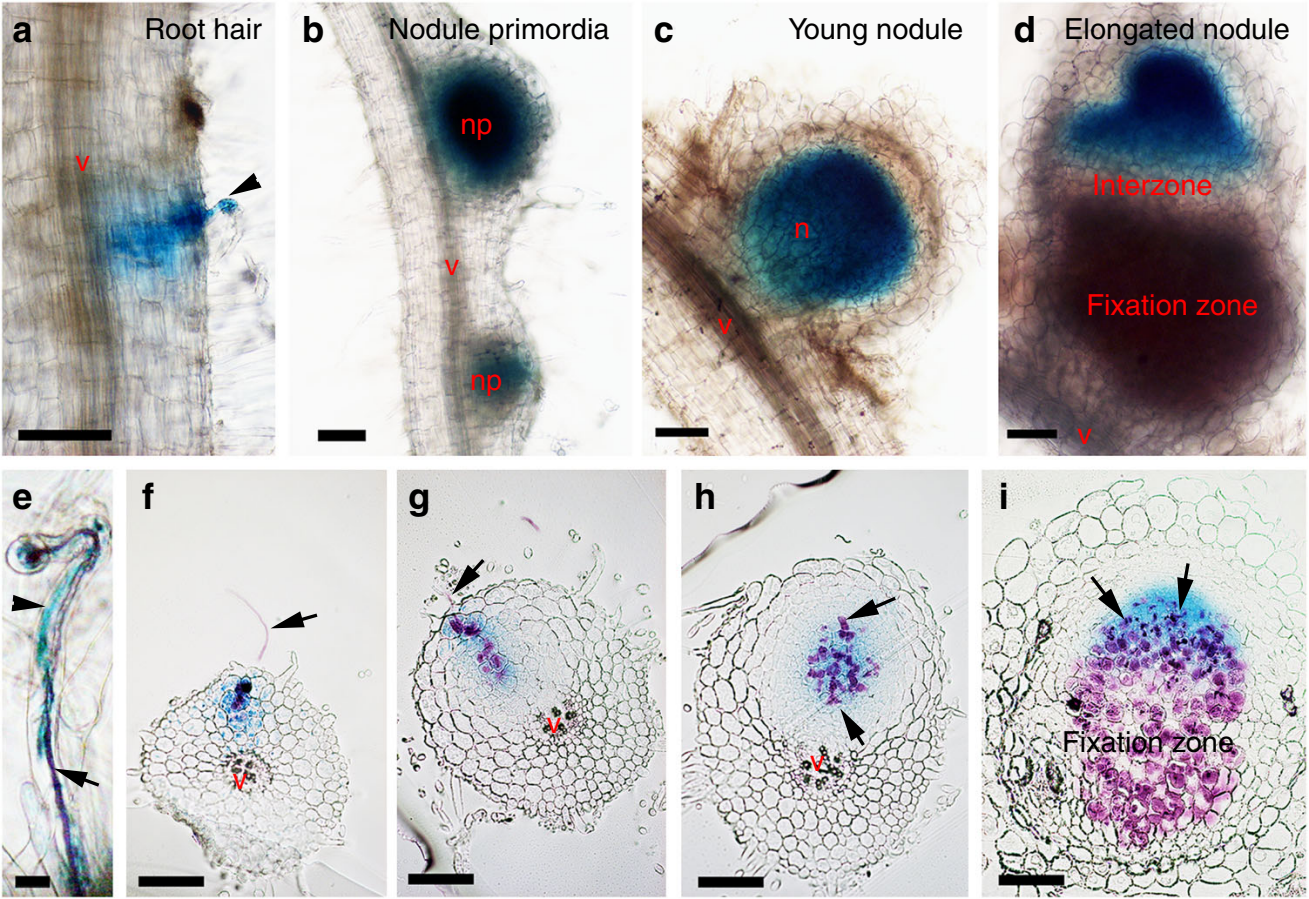
Expression of *VPY* is tightly associated with rhizobial infection in root and nodule cells. **a**–**i** Images showing *pVPY:GUS* activity during rhizobial infection in root hair (**a**, **e**) and in nodules from primordia to mature zonation stages (**b**–**d**, **f**–**i**). Composite transgenic plants were used at different stages after inoculation with Rm1021-lacZ. **a**–**e** are whole mount of GUS and/or X-gal stained roots, **f**–**i** are longitudinal sections of GUS and X-gal stained nodule primordia/nodule. Blue colour indicates staining of GUS activity and magenta colour indicates staining of rhizobia. Arrowheads indicate GUS stained root hairs (**a**, **e**) and arrows indicate infection threads in root hairs (**a**, **e**) or nodule primordia (**f**–**h**)/nodule (**i**). v, central vasculature of the root; np, nodule primordium; n, nodule. Scale bars, 100 µm

The expression of *VPY* in *M. truncatula* root hairs is induced by both Nod factors and *S. meliloti*^48^. We further determined the spatiotemporal pattern of *VPY* expression using a fusion of the *VPY* promoter to the *GUS* gene (*pVPY:GUS*) in composite plants. GUS staining revealed that reporter activity was highest in the root hairs harbouring either microcolonies or developing infection threads (Fig. 2a,  e, Supplementary Fig. 2a–b). During nodule development, GUS activity was found in young developing nodules and at the early symbiotic region of elongated nodules, but not in the interzone or nitrogen fixation zone (Fig. 2b–d). Longitudinal sectioning showed that the GUS staining was confined to the group of cells harbouring or surrounding infection threads (Fig. 2f–h). Later, after zonation of nodules, the expression of *VPY* was mainly found in the infection zone (Fig. 2i). Thus, *VPY* expression is correlated with active infection during both epidermal and nodule primordia infection as well as in mature nodules and is therefore coherent with the defective rhizobial infections observed in both root hairs and nodule cells in *vpy* mutants.

### Localization of VPY during rhizobial infection

**Fig. 3:**
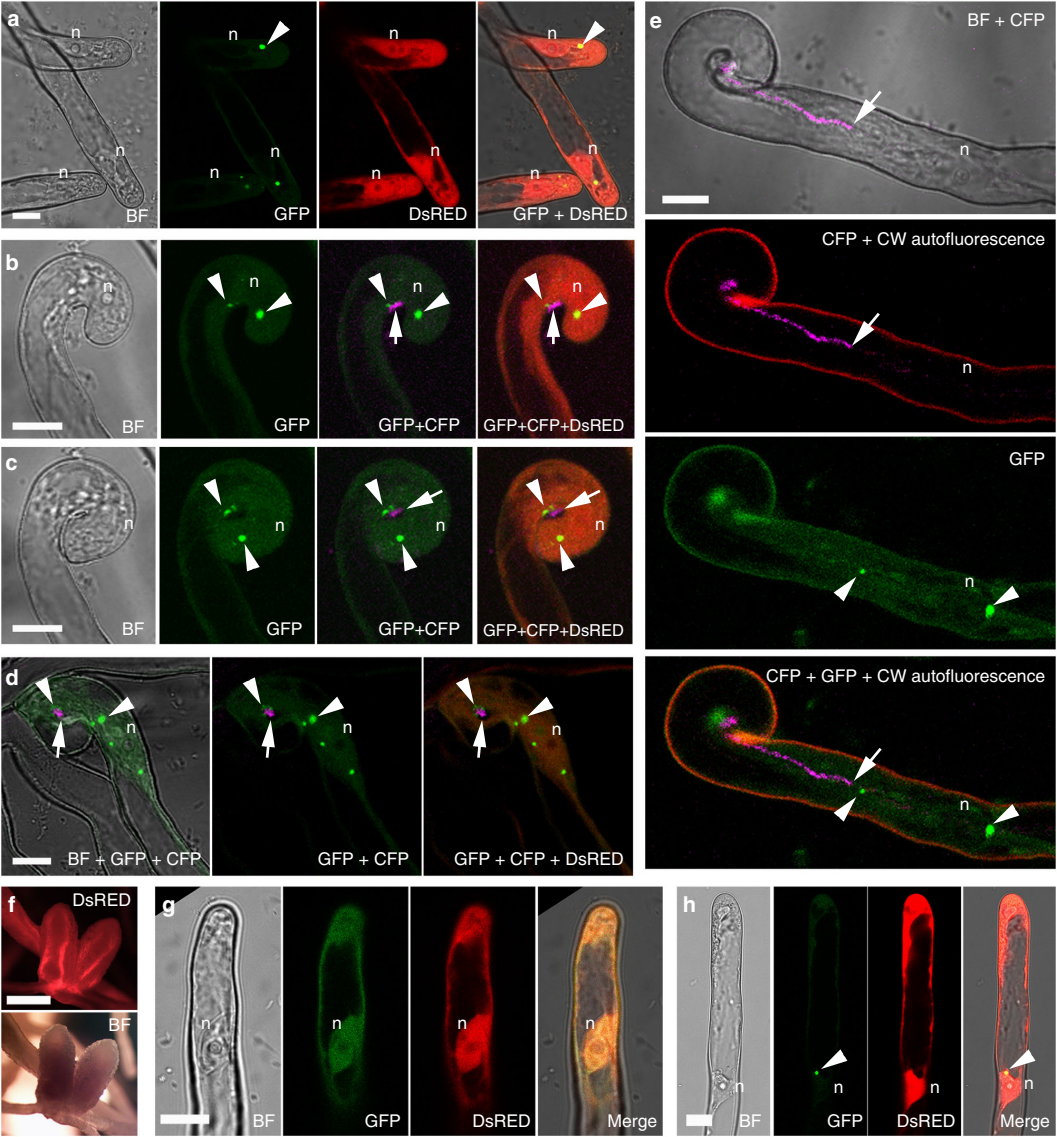
Subcellular localization of VPY during rhizobial infection. **a**–**e** Live cell confocal images showing localization of VPY-GFP driven by *VPY* promoter in root hairs at different stages of rhizobial infection. The VPY-GFP fusion is observed in small dot-like bodies, in the vicinity of the nucleus, in elongating root hairs prior to deformation (**a**). During root hair curling around attached rhizobia and bacteria entrapment by curl closure, VPY-GFP labelled puncta are preferentially found close by the nucleus and in front of rhizobial attachment point (**b**, **c**), a localization pattern that persists during infection chamber remodelling (**d**); Finally, when polar elongation of the infection thread starts, one VPY-GFP punctus  is found at   the growing tip of the infection thread, situated a few micrometers ahead of the colonizing rhizobia (**e**). Images are representative of more than 100 root hairs from at least 20 A17 composite plants (**a**), more than 50 root hairs imaged in eight different *sunn-2* composite plants (**b**–**d**) and more than 40 ongoing infection sites, monitored in 15 *sunn-2* composite plants (**e**) in three independent experiments. **f** Nodules from transgenic *vpy-2* plants complemented by *pVPY:VPY-GFP* construct. **g**, **h** Subcellular localization of MSP domain or ankyrin repeats containing domain of VPY in plants transformed with *pLjUBQ1: GFP-MSP* (**g**) *or pLjUBQ1: GFP-ankyrin* (**h**). All constructs contain a constitutively expressed DsRED marker (red colour). Root hairs were imaged from 2 dpi up to 7 dpi with Rm1021-CFP (**a**, **g** and **h**) or Sm2011-CFP (**b**–**e**) and nodules were examined at 60 dpi. Bright Field (BF), DsRED, GFP and CFP images were pseudo coloured in grey, red, green and magenta respectively, except in **e** where wall autofluorescence is in red. Plants in **a**, **g** and **h** are in WT A17 background. Plants in **b**–**e** are in *sunn-2* background. n, nucleus. Arrowheads indicate GFP puncta. Arrows indicate CFP labelled rhizobia. Scale bars, 10 µm (**a**–**e**, **g**–**h**) and 1000 µm (**f**)

The subcellular localization of VPY in root hairs was investigated in detail by live cell confocal imaging in roots of A17 or *sunn-2* expressing a *pVPY:VPY-GFP* construct. Expression of this construct in *vpy* roots restored nodulation (about 30 nodules per plant 30 dpi with *S. meliloti*), indicating that fusion with GFP does not affect VPY function (Fig. 3f, Supplementary Fig. 3a). Using live cell confocal imaging, we were able to follow the dynamics of VPY-GFP throughout the root hair infection process (Fig. 3a–e). Following rhizobial inoculation, VPY-GFP localized to small puncta in the cytoplasm of most root hairs, notably growing root hairs (Fig. 3a). These puncta, although generally static, were occasionally mobile and were most often found in the vicinity of the nucleus (Fig. 3a; Supplementary Video 1). In curling root hairs, the VPY-GFP labelled puncta were similarly present in the vicinity of the nucleus, and in addition, several puncta were concentrated in a position facing the attached rhizobia, before and after curl closure (Fig. 3b, c) as well as during infection chamber development (Fig. 3d). In the root hairs harbouring actively growing infection threads, VPY-GFP was consistently found to accumulate at the growing tip of the infection thread and in nuclear-associated puncta (Fig. 3e; Supplementary Fig. 4). Closer examination of the infection thread tip-localized fluorescence showed that VPY-GFP accumulates in a highly dynamic assembly capping the growing infection thread tip, and sometimes extending into the cytoplasm (Supplementary Video 2). In the absence of rhizobia, VPY-GFP localized uniformly to the cytoplasm and occasionally a weak GFP signal was found in the nucleus (Supplementary Fig. 5). The weak GFP signal is consistent with the weak expression of *VPY* in root hairs before rhizobial inoculation^42,48^. This background localization was often observed in inoculated roots (Fig. 3) in addition to the specific punctate localization (Fig. 3). However, VPY-GFP was not observed in puncta in non-inoculated roots (Supplementary Fig. 5), suggesting that accumulation of VPY-GFP in small subcellular domains is specifically induced following rhizobial inoculation. Altogether, our results indicate that, in response to inoculation, VPY accumulates in small cytoplasmic domains of unknown nature associated mainly with the nucleus and the apical tips of growing infection threads. This localization correlates well with the *vpy* mutant phenotypes, further suggesting a role of VPY in polarized infection thread construction and related secretion.

To determine which domain of the VPY protein is responsible for the punctate localization, constructs were made to express GFP fused either to the N-terminal MSP domain (GFP-MSP) or to the C-terminal ankyrin repeat-containing domain (GFP-ankyrin) of VPY, both driven by the strong *LjUBQ1* promoter in roots of composite plants. After rhizobial inoculation, GFP-ankyrin displayed the punctate pattern characteristic of full-length VPY, while GFP-MSP was found uniformly distributed in the cytoplasm and nucleus (Fig. 3g, h), arguing that the ankyrin repeats are responsible for the localization of VPY to the punctate, rhizobia inoculation-induced subdomains.

### LIN and VPY co-localize during rhizobial infection

**Fig. 4:**
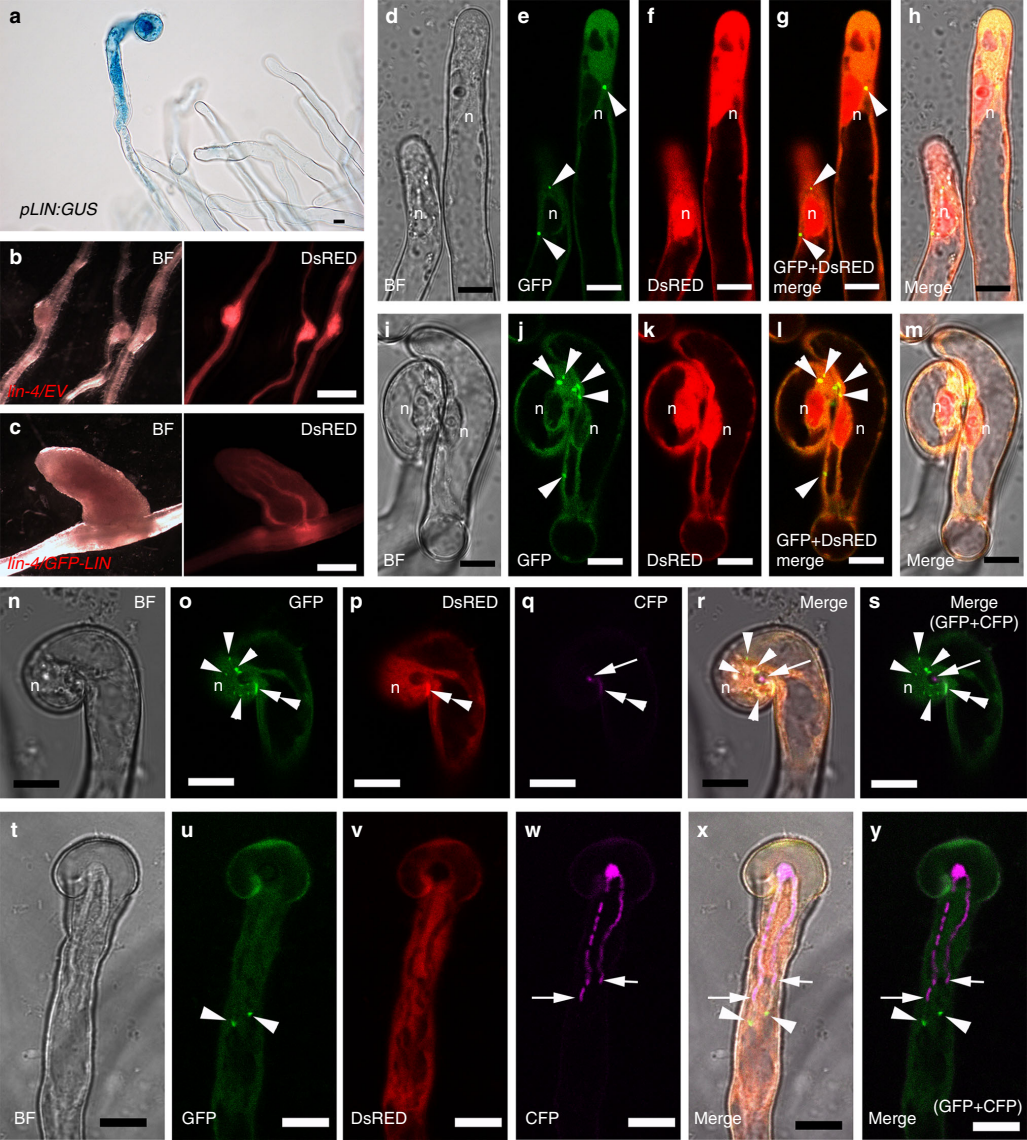
Expression of *LIN* and subcellular localization of LIN in root hairs during rhizobial infection. **a** Expression of *pLIN:GUS* in a root hair harbouring an infection thread. **b**–**c** Nodules from *lumpy infections-4* (*lin-4*) mutants transformed with an empty vector (EV) (**b**) or *pLIN:GFP-LIN* construct (**c**). Bright field (BF), left and DsRED images, right in **b** and **c**. **d**–**y** Live cell confocal images of subcellular localization of GFP-LIN driven by *pLjUBQ1* promoter in root hairs throughout different stages of rhizobial infection: GFP-LIN accumulated in puncta associated to the nuclear periphery in non-deformed root hairs (**d**–**h**); GFP-LIN labelled dot-like bodies were also found around rhizobial micro-colony and neighboring nucleus during infection chamber development (**i**–**m**, **n**–**s**); and finally, GFP-LIN also accumulated at the tip of infection threads, just ahead of the elongating structure (**t**–**y**). DsRED was used as a transgenic marker (red). Transgenic plants were either inoculated with Rm1021-lacZ (**a–c**) or Rm1021-CFP (**d**–**y**, magenta in **q**–**s**, **w**–**y**). Plants were imaged from 2 dpi up to 7 dpi (**d**–**y**). GUS staining is shown as blue. DsRED, GFP, CFP were pseudo coloured in red, green and magenta, respectively. n, nucleus. Arrowheads, GFP-LIN puncta. Arrows, CFP tagged rhizobia. Double arrowheads, auto-fluorescence. Scale bars, 10 µm (**a**, **d**–**y**) and 500 µm (**b**, **c**)

The MSP domain and ankyrin repeats of VPY are both predicted to mediate protein-protein interactions, suggesting that VPY function in rhizobial infection may involve interactions with one or more other proteins. We have noticed that *VPY* exhibits striking similarities in terms of symbiotic phenotypes and expression patterns with *LIN*. Both *vpy* and *lin* show delayed initiation and development of infection threads and both of them form aberrant nodule-like structures with a characteristic central vasculature^40,42,47,49^. *LIN* and *VPY* also have similar expression patterns in nodules^40^ (Fig. 2). And when a construct containing the *LIN* promoter driving the GUS reporter gene (*pLIN:GUS*) was expressed in *M. truncatula*, strong GUS activity was detected in root hairs harbouring infection threads (Fig. 4a), reminiscent of *VPY* expression in root hairs (Fig. 2a, e). Based on these similarities we investigated the possibility that VPY and LIN might act together during rhizobial infection.

**Fig. 5:**
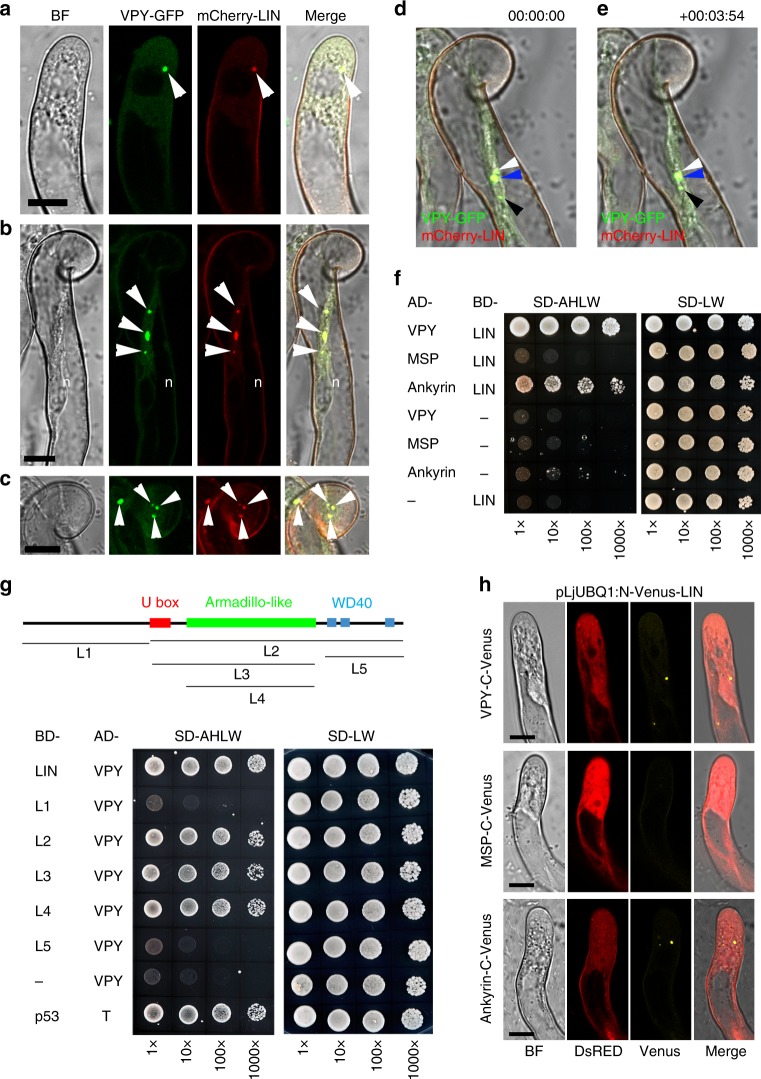
Interaction of VPY and LIN. **a**–**c** Live cell confocal images of root hairs from transgenic plants containing *pVPY:VPY-GFP* and *pLjUBQ1:mCherry-LIN* showing co-localization of VPY and LIN after inoculation with rhizobia. **d**, **e** Live cell images from the root hair tip shown in **b**, **c** at two time points (0″ and 3′54″) showing the movement and fusion of VPY-GFP/mCherry-LIN labeled puncta. **f**–**g** Yeast two-hybrid assays: between LIN and VPY, MSP domain of VPY and ankyrin repeats of VPY, respectively (**f**) and between VPY and different domains of LIN (**g**). The combination of proteins expressed in either the AD vector (pGADT7) or the BD vector (pGBKT7) are indicated alongside the yeast colonies. Diploid yeast cells with a series of 10- fold dilutions were grown on either SD-Leu-Trp (SD-LW) or SD-Ade-His-Leu-Trp (SD-AHLW) media. **h** Bimolecular Fluorescence Complementation (BiFC) experiments after inoculation with rhizobia. Split Venus fluorescent protein was used, with N-Venus fused to LIN driven by *pLjUBQ1* promoter and C-Venus fused to VPY, MSP domain or ankyrin repeats containing domain of VPY driven by *VPY* promoter. DsRED was used as a transgenic marker. DsRED and GFP images were pseudo coloured in red and green, respectively. n, nucleus; Arrowheads, VPY-GFP/mCherry-LIN puncta. Scale bars, 10 µm

Firstly, the sub-cellular localization of LIN was investigated by making use of an N-terminal fusion to GFP driven by either the *LjUBQ1* promoter or the native *LIN* promoter. Both *pLIN:GFP-LIN* and *pLjUBQ1:GFP-LIN* complemented *lin* mutants (Fig. 4b, c; Supplementary Fig. 3; Supplementary Fig. 6). Live cell imaging of the roots of composite plants transformed with either construct and then inoculated with *S. meliloti*, showed similar localization patterns (Fig. 4; Supplementary Fig. 7). Since the fluorescent signal was much stronger for *pLjUBQ1:GFP-LIN*, this construct was used for subsequent experiments. No GFP signal for *pLjUBQ1:GFP-LIN* was observed under non-symbiotic conditions in either root hairs or roots, suggesting tight control of LIN accumulation at the protein level. When root hairs were imaged in *S. meliloti*-inoculated roots, GFP-LIN localized primarily to a few puncta in close proximity to the nucleus, while weaker GFP fluorescence was visible throughout the cytoplasm (Fig. 4d–h). After bacterial entrapment within curled root hairs or between touching root hairs, puncta were enriched around the developing infection chamber and the adjacent nucleus (Fig. 4i–s). Finally, in root hairs with growing infection threads, GFP-LIN accumulated in the cytoplasm at the very tip of infection threads (Fig. 4t–y) with some puncta also found close to the nucleus in the same root hair (Supplementary Fig. 8). This localization of LIN during rhizobial root hair infection, highly reminiscent of that observed for VPY, prompted us to test whether these two proteins co-localize. For this, *M. truncatula* composite plant roots were generated using a construct containing both *pVPY:VPY-GFP* and *pLjUBQ1:mCherry-LIN*. Live-cell imaging of root hairs in *S. meliloti*-inoculated roots showed that VPY-GFP co-localized perfectly with mCherry-LIN within the puncta and the cytoplasm (Fig. 5a–e).

### VPY interacts with LIN in yeast and *in planta*

**Fig. 6:**
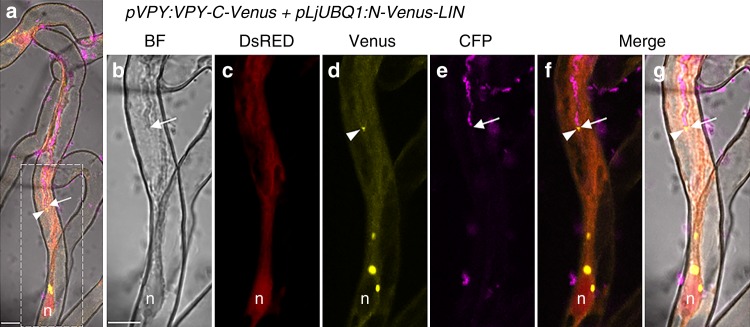
VPY interacts with LIN during rhizobial infection thread formation. **a**–**g** Live cell confocal images showing a root hair undergoing infection in a *sunn-2* composite plant containing *pVPY: VPY-C-Venus*, *pLjUBQ1:N-Venus-LIN* and *pAtUBQ:DsRED* as a transgenic marker. Bimolecular Fluorescence Complementation (BiFC) experiments were performed using N-Venus fused to LIN driven by *pLjUBQ1* promoter and C-Venus fused to VPY driven by *VPY* promoter as in Fig. 5h. One representative image is shown out of seven imaged ongoing infection sites in two *sunn-2* composite plants. Split Venus fluorescence was imaged 4 days after inoculation with Sm2011-CFP, in a root hair hosting an elongating infection thread. Venus fluorescence was associated notably to the tip of the growing infection thread. As in other root hairs, Venus fluorescence was also found associated to a number of other puncta in the nucleus vicinity or along the cytoplasmic bridge. DsRED, Venus and CFP were pseudo coloured in red, yellow and magenta respectively. Dashed white line box in **a** indicates the regions shown in (**b**–**g**). Arrows, infection thread growing tip; arrowheads, infection thread tip-associated Venus-labelled puncta. n, nucleus. Scale bars, 10 µm

By using a yeast two-hybrid assay we were able to show that a VPY prey could interact with LIN when used as a bait (Fig. 5f). Additional yeast interaction assays indicated that LIN interacted with the VPY C-terminal fragment containing the ankyrin-repeat domain but not with one containing the N-terminal MSP domain (Fig. 5f). LIN is a putative E3 ubiquitin ligase with an unknown N-terminal domain, a U-box, an Armadillo-like domain and a C-terminal domain containing WD40 repeats^40^. Yeast two-hybrid tests using truncated LIN with different domain combinations showed that only the Armadillo-like domain was indispensable for the interaction with VPY (Fig. 5g). The interaction between LIN and VPY was further evaluated using Bimolecular Fluorescence Complementation (BiFC)^50^ in *M. truncatula*, using C-terminal and N-terminal split-Venus fragments fused to VPY and LIN respectively. N-Venus-LIN was expressed from a strong constitutive promoter (*pLjUBQ1:N-Venus-LIN*) to improve detection and VPY-C-Venus was driven by the native *VPY* promoter (*pVPY:VPY-C-Venus*) to better reflect natural conditions. Strong Venus fluorescence was found in puncta in non-deformed root hairs (Fig. 5h) as well as curled root hairs (Supplementary Fig. 9) imaged in *S. meliloti*-inoculated composite plants expressing the BiFC fusions at 7 dpi, thus showing that VPY and LIN interact *in planta* following rhizobial inoculation. In root hairs undergoing rhizobial infection Venus fluorescence was also associated with the growing infection thread tip (Fig. 6). The small puncta labelled by the interacting proteins were alike in every aspect to those labelled by either GFP-LIN or VPY-GFP at identical stages (Figs. 2, 3). Additional BiFC experiments using N- or C-terminal truncated versions of VPY showed that the C-terminal ankyrin repeat-containing domain interacted with LIN, whereas the N-terminal MSP domain on its own did not (Fig. 5h), consistent with the yeast two-hybrid results and the localization of GFP-ankyrin (Figs. 5f, 3h). We therefore conclude that VPY interacts with LIN in rhizobia-inoculated plants, and that this interaction and its localization to puncta requires the C-terminal ankyrin repeat-containing domain.

### VPY is regulated by LIN during rhizobial infection

**Fig. 7:**
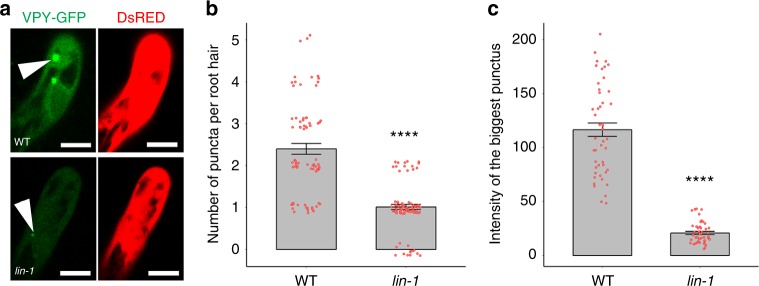
Regulation of VPY by LIN. **a** Confocal images of VPY-GFP driven by *VPY* promoter in root hairs of wild type (WT) A17 or *lin-1* mutant under the same condition at 5 dpi with Rm1021-CFP. DsRED was used as a transgenic marker. DsRED and GFP were pseudo coloured in red and green respectively. Scale bars, 10 µm. **b**, **c** Quantification of the numbers of VPY-GFP puncta per root hair (**b**), and integrated fluorescence intensity of the brightest punctus (**c**) in wild type (WT) A17 and *lin-1* mutant. Bars indicate standard error. ****p < 0.0001, Student’s *t-*test. *n* = 75 (WT) and 84 (*lin-1*) root hairs for (b) and *n* = 47 (WT) and 46 (*lin-1*) for (c) Source data are provided as a Source Data file

Protein ubiquitination has diverse functions and can affect the degradation, sub-cellular localization, activity or interaction of proteins^51^. The observed interaction of VPY and LIN led us to monitor whether VPY localization is dependent on LIN. To answer this, confocal imaging was used to test *pVPY:VPY-GFP* localization in the *lin-1* mutant inoculated with *S. meliloti*. Compared with wild type, the VPY-GFP signal was much reduced in *lin-1*, both in the puncta and the cytoplasm (Fig. 7a). In addition, quantification of the average number of VPY-GFP puncta per root hair revealed a >2 fold decrease in *lin-1* compared to WT (Fig. 7b). This could be due to a genuine decrease in the number of labelled puncta, or from the difficulty of detecting puncta with lower fluorescence levels. To distinguish between these two possibilities, the fluorescence intensity of the largest punctus per root hair was determined for both *lin* and WT plants. We found that the intensity of the brightest punctus was greatly reduced in *lin* (Fig. 7c). To determine whether the observed reduction in VPY levels was a consequence of reduced *VPY* expression in the *lin* mutant we introduced *pVPY:GUS* into wild type and *lin-1* backgrounds. We found similar GUS staining in infected root hairs for both genotypes suggesting that regulation of VPY by LIN is at posttranscriptional level (Supplementary Fig. 10). This, along with the finding that cytoplasmic VPY levels also appeared to be decreased in *lin*, suggests a role for LIN in the regulation of VPY abundance or stability, rather than its characteristic punctate localization. This positive regulation of VPY by LIN is in line with the duo’s positive roles during rhizobial infection. To test whether VPY could affect the subcellular localization of LIN, we introduced *pLjUBQ1:GFP-LIN* into *vpy-1* mutant and we found persistent punctate localization of LIN-GFP, as observed in wild type (Supplementary Fig. 11).

### *VPY-LIKE* and *LIN-LIKE* may contribute to rhizobial infection

**Fig. 8:**
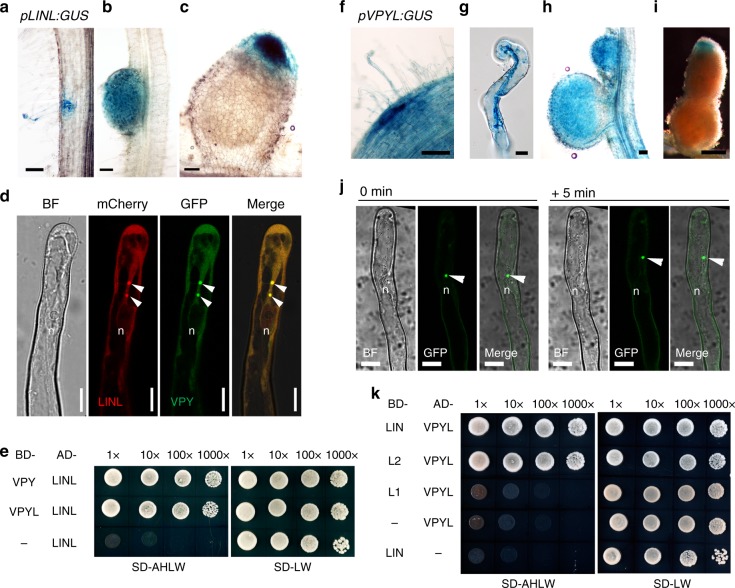
A protein complex containing VPY, VPYL, LIN and LINL. **a**–**c** Expression of *LINL* in an infected root hair (**a**), in a young nodule (**b**) and in a mature nodule (**c**) as shown by *pLINL:GUS* activity. **d** Co-localization of VPY (green) and LINL (red) in root hairs of rhizobia-inoculated hairy roots transformed with *pVPY:VPY-GFP* / *pLjUBQ1:mCherry-LINL*. **e** Yeast two-hybrid assays between VPY, VPYL and LINL. **f**–**i** Expression of *pVPYL:GUS* at infection site (**f**), in a root hair harbouring an infection thread (**g**), young and mature nodules (**h**, **i**). **j** Live cell images of localization of GFP-VPYL driven by *pLjUBQ1* promoter at two time points (0 min and 5 mins later) in a root hair after inoculation with rhizobia. **k** Yeast two-hybrid assays between VPYL and LIN, C terminus of LIN (L2) and N terminus of LIN (L1). BD, pGBKT7; AD, pGADT7. SD-LW, SD-Leu-Trp; SD-AHLW, SD-Ade-His-Leu-Trp. GUS staining were shown as blue. DsRED and GFP were pseudo coloured in red and green respectively. Arrowheads, GFP/mCherry puncta. n, nucleus. Scale bars, 100 µm (**a**–**c**, **f,** **h**, **i**) and 10 µm (**d**, **g** and **j**)

The defective but not abolished rhizobial infection of *vpy* and *lin* could result from genetic redundancy. Indeed, close homologs exist in *M. truncatula* for both *LIN*, which we have named *LIN-LIKE* (*LINL*; Medtr8g103227), and for *VPY*, called *VPY-LIKE* (*VPYL*)^43^ (Supplementary Fig. 12; Supplementary Fig. 13). We therefore investigated their expression during nodulation using promoter-GUS constructs (*pLINL:GUS* and *pVPYL:GUS*) in composite plants. Similarly to *LIN* and *VPY* fusions, the *pLINL:GUS* reporter also conferred high GUS activity levels in infected root hairs, in young nodules, and at the apical region of elongated nodules (Fig. 8a–c). This suggests that *LINL* may also contribute to rhizobial infection. In contrast, the *pVPYL:GUS* fusion was constitutively expressed throughout the root (Fig. 8f–h), including both infected and non-infected root hairs (Fig. 8f, g), consistent with our earlier published root hair infectome data^48^. *VPYL* was also expressed in young nodules and the apex of mature nodules (Fig. 8h, i).

We then tested the sub-cellular localization of LINL (*pLjUBQ1:mCherry-LINL*) together with VPY (*pVPY:VPY-GFP*). mCherry-LINL localized to puncta and cytoplasm in root hairs of transgenic composite plants inoculated with *S. meliloti*, and entirely co-localized with VPY-GFP (Fig. 8d). GFP-VPYL (*pLjUBQ1:GFP-VPYL*) showed a similar punctate pattern, and, as in the case of VPY-GFP, certain puncta were mobile (Fig. 8j). We further tested the interaction of VPY and LINL using a yeast two-hybrid assay and found that they were indeed able to interact (Fig. 8e). Further yeast two-hybrid tests showed that VPYL interacted with both full-length LIN and the C-terminus of LIN (Fig. 8k). Finally, VPYL also interacted with LINL in yeast (Fig. 8e).

Taken together, the gene expression, subcellular localization and protein-protein interaction data obtained here suggest that LINL and VPYL may act together with LIN and VPY to promote rhizobial infection. We therefore propose that these proteins form complexes within punctate bodies in the cytoplasm and at the tip of growing infection threads.

### An exocyst subunit co-localizes with the VPY-LIN complex

**Fig. 9:**
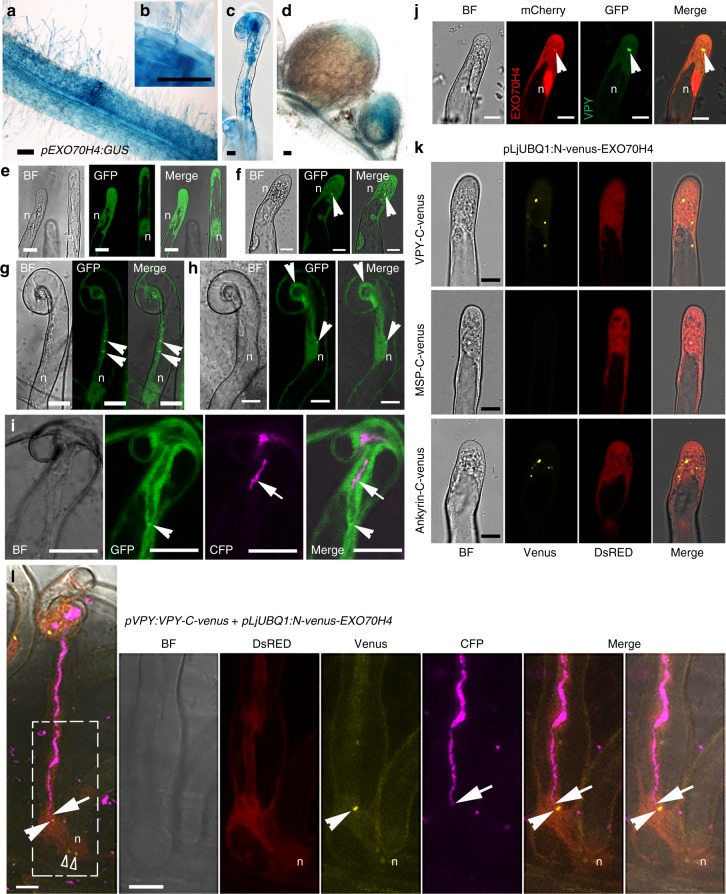
EXO70H4 co-localizes with VPY. **a**–**d** Expression of *EXO70H4* in roots inoculated by rhizobia (**a**), at a rhizobial infection site (**b**), in a root hair containing an infection thread (**c**) and in nodules (**d**) as shown by *pEXO70H4:GUS* activity. **e** Subcellular localization of GFP-EXO70H4 in root hairs without inoculation with rhizobia. **f**–**i** Subcellular localization of GFP-EXO70H4 in root hairs following inoculation with Rm1021-CFP (magenta). **j** Co-localization of VPY-GFP (green) and mCherry-EXO70H4 (red) at the same punctus in root hairs of rhizobia-inoculated composite plants transformed with *pVPY:VPY-GFP* / *pLjUBQ1:mCherry-EXO70H4*. **k** Bimolecular Fluorescence Complementation (BiFC) experiments in root hairs following inoculation with rhizobia. Split Venus fluorescent protein was used, with N-Venus fused to EXO70H4 driven by *pLjUBQ1* promoter and C-Venus fused to VPY, MSP domain or ankyrin repeats containing domain of VPY driven by *VPY* promoter. **l** Live cell confocal images showing a root hair undergoing infection in a *sunn-2* composite plant containing *pVPY:VPY-C-Venus*, and *pLjUBQ1:N-Venus-EXO70H4*. Split Venus fluorescence was imaged 3d after inoculation with Sm2011-CFP, in a root hair hosting an elongating infection thread. Venus fluorescence (arrowheads in **l**) was associated notably to the tip of a growing infection thread (arrows); Venus fluorescence (open arrowheads) was also found associated to a number of other puncta in the nucleus vicinity. Five ongoing infection sites where imaged in four plants in **l**. GUS staining is shown as blue. DsRED was used as a transgenic marker. DsRED, Venus, GFP and CFP were given pseudo coloured in red, yellow, green and magenta respectively. n, nucleus. Arrowheads, GFP/mCherry /Venus puncta. Arrows, CFP tagged rhizobia. Scale bars, 100 µm (**a**, **d**) and 10 µm (**b**, **c**, **e**–**l**)

VPY is required for epidermal entry of AM fungi and arbuscule development during mycorrhization^42–44^. In addition, it has been shown that an exocyst component, EXO70I, is involved in mycorrhization and partially co-localizes with VPY in mycorrhizal roots of *M. truncatula* but is dispensable for nodulation^46^. Our previous transcriptome profiling in *M. truncatula* shows that another *EXO70* family member, *EXO70H4* (Medtr4g062330) (Supplementary Fig. 14), is induced 3 and 5 dpi in root hairs after inoculation with rhizobia^48^ (Supplementary Fig 15). A *pEXO70H4:GUS* fusion revealed that *EXO70H4* was constitutively expressed in *Medicago* roots, especially in root tips and lateral root primordia (Supplementary Fig. 16). After inoculation with rhizobia, *EXO70H4* expression was enhanced in infected root hairs and the underlying cortical cells (Fig. 9a–c). *EXO70H4* expression was also detected in young nodules, and in the apical region of elongated nodules, including the infection zone (Fig. 9d). Thus, the overall expression pattern of *EXO70H4* is consistent with a potential contribution of the encoded exocyst component to rhizobial infection.

The localization of EXO70H4 was then investigated by live-cell imaging. Since no GFP signal was detected using the native *EXO70H4* promoter driving expression of a *GFP-EXO70H4* gene fusion, we instead used the *LjUBQ1* promoter. A strong GFP-EXO70H4 signal was detected in the cytoplasm and the nucleus under non-symbiotic conditions in root hairs and other root cells (Fig. 9e, Supplementary Fig. 17). After rhizobial inoculation, in addition to fluorescence in the cytoplasm and the nucleus, GFP-EXO70H4 was highly enriched in a small number of root hair puncta (Fig. 9f). In curled root hairs, puncta were also found close to the nucleus, along the cytoplasmic bridge between the infection chamber and the nucleus, and close to the infection chamber (Fig. 9g, h). In root hairs hosting an infection thread, GFP-EXO70H4 accumulated at the infection thread tip (Fig. 9i), reminiscent of the complex labelled by VPY-GFP (Fig. 3e) and GFP-LIN (Fig. 4t–y).

The relative localization of EXO70H4 and VPY was investigated using a construct expressing *pLjUBQ1:mCherry-EXO70H4* and *pVPY:VPY-GFP*. Upon rhizobial inoculation mCherry-EXO70H4 was found in the cytoplasm and nucleus and was highly enriched in puncta, where it co-localized with VPY-GFP (Fig. 9j). Next we did BiFC with the combination of *pVPY:VPY-C-Venus* and *pLjUBQ1:N-Venus-EXO70H4* constructs. Strong Venus fluorescence was observed in cytoplasmic puncta in *M. truncatula* root hairs inoculated with *S. meliloti* (Fig. 9k). Using truncated VPY proteins containing either the N- or C-terminal domains, we again found that only the ankyrin repeat-containing domain was able to show Venus fluorescence puncta with EXO70H4 (Fig. 9k). Finally, strong Venus fluorescence was once more associated with the tip of the growing infection thread (Fig. 9l). Altogether, these results indicate that EXO70H4 is systematically co-localized with VPY and suggest a possible role for EXO70H4 as an additional component of the protein complex comprising VPY and LIN.

### *EXO70H4* is required for rhizobial infection at early stages

**Fig. 10:**
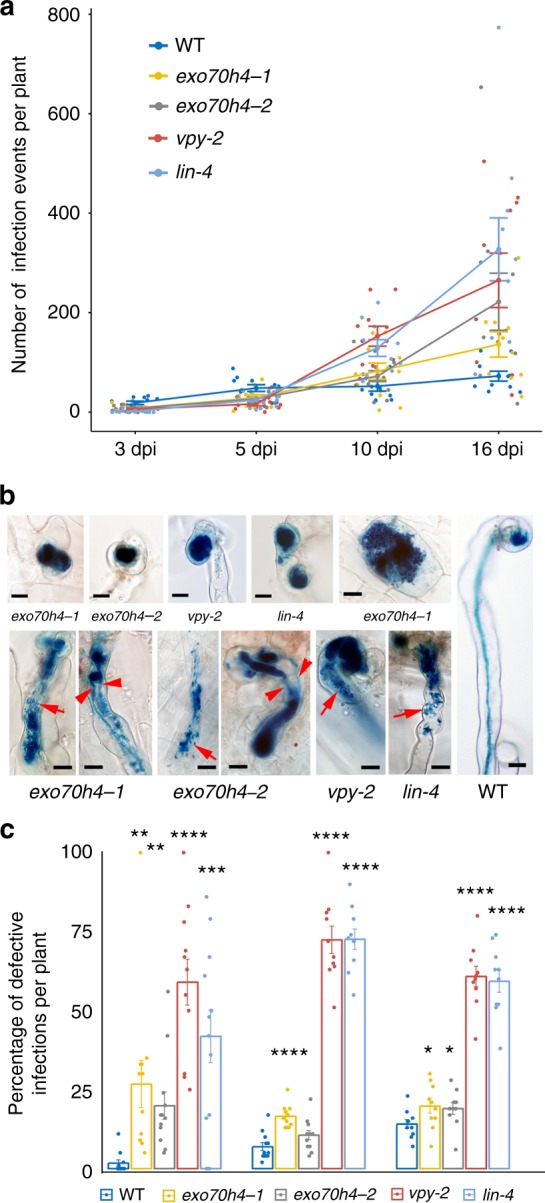
Rhizobial infection phenotypes of *exo70h4, vpy* and *lin*. **a** Rhizobial infection time course of WT, *exo70h4-1*, *exo70h4-2*, *vpy-2* and *lin-4*. All infection events from microcolony to ramified cortical infection threads were counted from 9 to 12 plants for each genotype at 3, 5, 10 and 16 dpi with Rm1021-lacZ respectively. Bars represent standard error. **b** Typical defective infection events of *exo70h4-1*, *exo70h4-2*, *vpy-2* and *lin-4* (left) and a normal elongating infection event of WT (right). Top left, abnormal enlarged microcolonies (*exo70h4-1*, *exo70h4-2*, *vpy-2* and *lin-4*) and blocked infection in the epidermal cells (*exo70h4-1*). Bottom left, blocked infection threads, branched infection threads (arrowheads) and disintegrated infection threads resulting in rhizobia release (arrows) in the mutant root hairs of *exo70h4-1*, *exo70h4-2*, *vpy-2* and *lin-4*. Scale bars, 10 µm. **c** Quantification of defective infection events in WT, *exo70h4-1*, *exo70h4-2*, *vpy-2* and *lin-4* at 5, 10, 16 dpi, respectively. All genotypes are in R108 background. Bars show the percentage of defective versus all infection events per plant. Error bars represent standard error. Two-tailed Student’s *t*-test, *p < 0.05; **p < 0.01; ***p < 0.001; ****p < 0.0001. Source data are provided as a Source Data file

To investigate whether *EXO70H4* is required for rhizobial infection, two *M. truncatula* lines (NF10274 and NF14802) with the *Nicotiana tabacum* transposable element *Tnt1* insertions in the single exon of *EXO70H4* were recovered from the Noble Research Institute mutant library^52^, designated as *exo70h4-1* and *exo70h4-2*, respectively (Supplementary Fig. 18). Homozygous mutants were isolated and infection phenotypes were characterized alongside *vpy-2* and *lin-4* grown in sand: terra green by histochemical staining of roots inoculated with a *Hem:LacZ* expressing strain of *S. meliloti* (Rm1021-LacZ). The *exo70h4-1* and *exo70h4-2* mutants displayed about 50% fewer infection events than wild type at 3 and 5 dpi (Fig. 10a). At 10 dpi, there was no significant difference between wild type and the *exo70h4* mutants, and at 16 dpi more infection events were present in both alleles of *exo70h4* compared to wild type (Fig. 10a). The dynamics of rhizobial infection of the *exo70h4* mutants were very similar to those of *vpy-2* and *lin-4*, both of which had fewer infections at early time points and more infections at later times by comparison with wild type (Fig. 10a). This pattern is likely the consequence of a feedback mechanism resulting from initial failed infections subsequently leading to increased infection attempts at later time points (Fig. 10a–c, Supplementary Fig. 18). Consistent with this, *exo70h4-1* and *exo70h4-2* showed many defective infections, such as oversized microcolonies and blocked infection threads that sometimes burst leading to the release of rhizobia within root hairs (Fig. 10b). Sometimes branched infection threads were observed in *exo70h4-1* and *exo70h4-2* (Fig. 10b). Similar phenotypes were also frequently found in *vpy-2* and *lin-4* mutants (Fig. 10b). Since occasionally “abnormal” infections could also be observed for the WT, we quantified all types of defective infection at 5, 10 and 16 dpi. We counted infection events at all stages from microcolony (MC) to ramified infection threads (rIT) into the cortex. For microcolonies, only big swelled ones were counted as “defective” while the small ones were counted as normal infection events. We found that the percentage of abnormal infections was higher in *exo70h4*, *vpy* and *lin* compared to wild type (Fig. 10c). However, there was no significant difference in nodule number between wild type and *exo70h4* mutants (Supplementary Fig. 18), consistent with the relatively mild infection phenotype. The phenotype of *exo70h4* mutants could be a result of genetic redundancy with members of the extensive EXO70 family (Supplementary Fig. 14), notably those which are also expressed in root hairs (Supplementary Fig. 15). Finally, in line with the lack of nodulation phenotype reported in Zhang et al. for the *exo70i* mutant^46^, we found no clear rhizobial infection phenotype for this mutant (Supplementary Fig. 19). Although the frequency of failed infections was much lower in *exo70h4* than in *lin* or *vpy*, the phenotypes of all three of these mutants were very similar in terms of the nature of the root hair infection defects and infection dynamics. We conclude that puncta-resident VPY and LIN most likely function together with EXO70H4 to promote rhizobial infection.

## Discussion

Nodulation involves two closely coordinated processes, nodule organogenesis and rhizobial infection^5^. While the main signalling pathway is well characterized in nodulation, the molecular and cellular mechanisms controlling rhizobial infection are far from clear^53^. Although a number of the components downstream of Nod factor perception and the common symbiosis signalling pathway have been identified, our knowledge about how these components function and cooperate to regulate rhizobial infection is limited. Here we report on the co-localisation and capacity to interact of two symbiosis-specific proteins, VPY and LIN, in root hairs of *M. truncatula* undergoing rhizobial infection. Furthermore, we have identified EXO70H4, a predicted subunit of the exocyst complex that regulates polar secretion and vesicle trafficking^54,55^, as an additional actor in rhizobial infection that, like VPY and LIN, is recruited to puncta associated to growing infection threads. *EXO70H4* is co-expressed with *VPY* and *LIN* in infected root hairs and the infection zone of the nodule and *exo70h4* mutants are partially defective in infection. The predicted function of EXO70H4 suggests a link of the VPY-LIN interaction module with the cell machinery for polarised secretion. The fact that the infection phenotypes of *vpy*, *lin* and *exo70h4* mutants are similar argues that a rhizobial infection-induced protein complex assembling VPY, LIN and possibly EXO70H4 is required for initiating and maintaining the polar growth of infection threads.

The development of a functional infection thread is essential for rhizobial colonization of the epidermal and outer cortical cell layers and the differentiating nodule tissues^3,27^. In *M. truncatula*, as in the majority of legumes, infection is preceded by entrapment of rhizobia within a closed root hair curl, followed firstly by infection chamber formation and then infection thread initiation^29^. These last two stages involve targeted exocytosis of cell wall and plasma membrane materials to the respective developing infection compartments, with a switch from isotropic growth of the infection chamber to polarised growth of the infection thread. Enlarged microcolonies are often found in *vpy, lin* and *exo70h4* mutants, suggesting that whilst exocytosis leading to infection chamber formation is unaffected, there is a defect in the initiation of the polar growth of the infection thread, as shown in detail for *vpy-1*^42^(Fig. 1). However, the existence of the close homologs *VPYL* and *LINL* make it difficult to rule out the possible involvement of the protein complex during infection chamber formation. Double mutants for each gene type will now be required to resolve this issue. Nonetheless, narrow elongating infection threads were rarely observed in mutants such as *vpy* and *lin*. Furthermore, we have shown that VPY, LIN and EXO70H4 localize to punctate subcellular foci in growing root hairs after rhizobial inoculation and accumulate precisely at sites along the cell wall of the infection chamber where infections threads will initiate (Figs. 3, 4 and 9). When an infection thread develops in the root hair the protein complex localizes at the very tip of the infection thread while one or more additional foci are always found loosely associated with the nucleus. The tip localized pattern is consistent with a role of VPY, LIN and EXO70H4 in orchestrating the focused exocytosis leading to tubular infection thread tip growth. The dual localisation of the protein complex at both ends of the cytoplasmic bridge linking the nucleus to the elongating infection thread tip further suggests a link of the complex with the polarisation of the cytoplasm between these two poles, possibly through interaction with the actin cytoskeleton. The defect in nuclear positioning in *vpy* root hairs after bacterial entrapment also supports a dual role of the infection protein complex both in maintaining an optimal distance between the developing infection compartment and the nucleus and in orientating the vesicle traffic. Both processes might involve the actin cytoskeleton. The relationships between the infection protein complex and actin organisation during rhizobial infection will have to be investigated in the future.

The bursting of certain infection threads in *vpy* and *lin* mutants and the large accumulation of matrix in the extracellular spaces of *vpy* nodules may also result from failure to correctly deliver extracellular materials or the delivery of incorrect or mistargeted materials to the infection compartment. Finally, the hyper-infection phenotypes in *vpy*, *lin* and *exo70h4* at late time points following rhizobial infection may reflect a general mechanism of impaired feedback regulation, leading to higher numbers of epidermal infections. Hyper-infection and enlarged microcolonies have also been found in other mutants such as *cbs1* and *npl*^33,39^, which are also required for the cell wall integrity of the infection thread.

VPY also localizes to punctate entities in AM fungal-colonized epidermal cells and arbuscule-containing cells during mycorrhization^43,44^. VPY homolog in petunia was reported in spherical structures associated with the vacuole, and thus termed “tonospheres”^44^. In rhizobial-infected root hairs, we found no clear association of puncta with the vacuole since these were present solely in cytoplasmic regions. It is conceivable that tonosphere bodies are present in infected root hairs but that the fluorescent signal is below the detection threshold. In the case of *M. truncatula*, LIN is not required for arbuscule development (Supplementary Fig. 20)^40^. However, *CERBERUS*, the *Lotus* ortholog of *LIN*, is induced during mycorrhization and AM fungal colonization is lower in *cerberus*^56,57^. Further studies on LIN and LINL during mycorrhization should help to elucidate whether a similar cellular mechanism operates during mycorrhizal infection and arbuscule development, as mycorrhization and nodulation not only share common signalling pathways but also exploit similar intracellular infection structures^14,58–61^.

The expansion of the EXO70 family in plants suggests functional diversification^62–66^. In *Arabidopsis* many EXO70s have been shown to be involved in different biological pathways, including roles for EXO70H proteins in cell wall maturation in trichomes and in plant-pathogen interactions^67,68^. Here we report that one *M. truncatula* EXO70 from the same H clade is required for symbiotic infection. It was recently reported that EXO70I, which belongs to a mycorrhizal-host specific clade, is also localized at the tip of the infection thread in nodule cells^69^. This suggests that multiple EXO70s have been recruited to symbiotic infection. Some other *EXO70s* are highly expressed in root hairs and some are slightly upregulated after rhizobial inoculation (Supplementary Fig. 15). It is likely that some of them may play redundant roles with EXO70H4 during rhizobial infection. Like EXO70s in pollen tubes^70^, different sets of exocyst complexes may also comprise distinct domains during tip growth in symbioses. Further supporting this is the finding that during mycorrhization an EXO84b from Arabidopsis localized as a crescent shape labelling the tips of intracellular hyphae and developing arbuscule branches in carrot roots^71^. Future studies should also reveal whether different exocyst subunits reside in the tip localized and nuclear-associated infection protein complex during rhizobial infection, as the scenario found for exocyst components during yeast budding^72^.

Our results show that VPY interacts with LIN through its series of C-terminal ankyrin repeats, consistent with our observation that the VPY C-terminus is necessary and sufficient for localization to the puncta. LIN is a putative E3 ligase containing U-box, Armadillo and WD repeat domains, and we have found that it interacts with VPY through the Armadillo domain. Decreased VPY-GFP levels in *lin* mutants suggests potential stabilization of the VPY protein by LIN. In one scenario, VPY may act as a scaffold protein facilitating access of LIN to its ubiquitination targets, rather than being itself a direct target for ubiquitination *per se*. In another scenario, LIN may ubiquitinate VPY in root hairs during rhizobial infection. However, considering the lower level of VPY in root hairs of *lin* after rhizobial inoculation, the function of ubiquitination therein is unlikely to be protein degradation. Ubiquitination of VPY could be required for its function as an adaptor, potentially recruiting EXO70H4, and thereby possibly the entire exocyst complex, to the rhizobial infection-induced protein complex at the tip of the infection thread. LIN may also regulate or even ubiquitinate EXO70H4, but the mode of its regulation there must be different from that of VPY, as EXO70H4 still showed similar levels and punctate localization in *lin* mutant after rhizobial inoculation (Supplementary Fig. 21). Though fewer in number, the VPY-labelled puncta are not completely abolished in *lin*, possibly due to the existence of LINL, which we show also co-localizes and interacts with VPY. Naturally, additional proteins can be expected to be part of the VPY-LIN protein complex, including other exocyst components and unknown proteins that could interact with the MSP domain of VPY and other domains of LIN. Future investigation on these proteins and their inter-regulations in the complex would further our understanding of the mechanisms underlying rhizobial infection.

In conclusion, our findings reveal an essential role of the protein complex comprising VPY and its interactors for intracellular infection via root hairs in *M. truncatula*. The presence of VPY and LIN orthologs in other legumes such as lupin^73^ suggests that the protein interaction module uncovered here may also be exploited during other types of rhizobial infection such as intercellular root entry.

## Methods

### Plant material and growth conditions

*M. truncatula* ecotype R108 or Jemalong A17 were used as wild type control. *sunn-2* is a γ-irradiation mutant in the Jemalong J5 background^74,75^. *sunn-2* has significantly more infection events compared to wild type, that are otherwise similar to those in wild-type plants. *vpy-1* is a fast neutron deletion mutant (FNB2) from the Jemalong A17 background^42^, and *vpy-2* is a *Tnt1* insertional mutant (NF6898) identified in the R108 background^42^. *lin-1* is an EMS mutant, from the Jemalong A17 background^40,49^. *lin-4* is a mutant with a point mutation characterized by forward screen from a *Tnt1* mutant population in the R108 background with a G to A substitution at position 1740bp of cDNA which creates a premature stop codon^47^. *exo70h4-1* (NF10274) and *exo70h4-2* (NF14802) are *Tnt1* insertional mutants in the R108 background described in this work. Seeds were treated with sulfuric acid for 10 mins and then washed with sterilized deionized water 6 times. The seeds were then treated with bleach for 4 mins, and then washed with sterilized deionized water six times. After imbibition in water for  >2 h, the seeds were transferred to water plates agar and left at 4 °C for two days, then at 22 °C overnight. Unless otherwise stated, the seedlings were transferred to a mixture of sand and terra green (1:1) (hereafter designated as sand:terra  green) and grown in a controlled environment chamber with 16 h/8 h light/dark photoperiod before inoculation with rhizobia.

### Rhizobium strains

Strains used in this study were *S. meliloti* strain Rm1021 (Rm1021) harbouring pXLGD4 (hemA:lacZ) (Rm1021-LacZ) or a pHC60^76^ derived plasmid conferring constitutive Cerulean CFP expression (Rm1021-CFP) as well as *S. meliloti 2011* harbouring pHC60 (Sm2011-GFP) or its CFP derivative (Sm2011-CFP).

### *Agrobacterium rhizogenes*-mediated transformation of Medicago

*Agrobacterium rhizogenes* ARqua1 strain was electroporated with binary vectors and used to generate composite plants comprising a transgenic hairy root system with non-transformed shoots and leaves^77^.

### Rhizobial infection and nodulation phenotyping

*Medicago* wild type R108, *vpy-2*, *lin-4*, *exo70h4-1* and *exo70h4-2* seedlings grown in sand:terra  green were inoculated with Rm1021-LacZ (overnight culture at 28 °C, OD_600_ = 0.02). Plants were harvested at 3, 5, 10, 16 dpi and the roots were fixed for 1 h in 2.5% glutaraldehyde then stained in 0.8 mg mL^−1^ X-GAL (5-Bromo-4-chloro-3-indolyl-ß-D-galactoside, Melford) solution with 100 mM sodium phosphate (pH 7.4), 10 mM KCl and 1 mM MgSO_4_ at 28 °C overnight in the dark. About 9–12 plants from each genotype were used for quantification of infection events at each time point. For the nodulation experiments, nodules from WT (R108) and *vpy* mutants were harvested at 47 dpi. Some nodules were stained in X-GAL and sectioned using a vibratome. For ultrathin sectioning, nodules were embedded in Technovit 7100 (Kulzer GmbH) resin following the manufacturer’s instructions and sectioned by using Ultramicrotome Leica EM UC7. The sections were then stained with 0.5% toluidine blue in 0.5% sodium tetraborate buffer for visualization of infection threads in nodules. For live cell phenotyping after rhizobial inoculation, *vpy-1* infection phenotype was compared to that of *sunn-*2 (10 plants for each genotype) in a live cell imaging set-up^30^. Ten infection sites were monitored in six different plants for each genotype.

### Promoter-GUS Assay

A 1260 bp *VPY* promoter was amplified by using HiFi Platinum Polymerase (Invitrogen) and ligated into pGEMT-easy vector (Promega) then cloned into pCAM2301 by *Xho*I and *Bgl*II to make *pVPY:GUS*. A 2287 bp *LIN* promoter was amplified by using Phusion High Fidelity Polymerase (NEB) and cloned into pDONR207 by using Gateway BP Clonase (Invitrogen). The BP product pDONR-pLIN was then cloned into the destination vector pKGWFS7 using Gateway LR Clonase (Invitrogen) to make *pLIN:GUS*. The *pVPYL:GUS*, *pLINL:GUS* and *pEXO70H4:GUS* constructs were made by Golden Gate cloning^78^. Promoters (*pVPYL*, 2932 bp; *pLINL*, 2780 bp; *pEXO70H4*, 1209 bp) were synthesized by Life Technologies and used as level 0 modules, then cloned into level 1 vector then level 2 vector EC50505 (https://www.ensa.ac.uk/). For the promoter-GUS analyses, more than 40 composite plants from each experiment were transferred to sand:terra green 4 weeks after transformation and inoculated with Rm1021-LacZ. The roots were harvested 3 weeks later for GUS staining and stained for at least 4 h at 37 °C in 1 mg ml^−1^ X-Gluc solution with 100 mM potassium phosphate buffer (pH7.0), 1 mM potassium ferricyanide, 1 mM potassium ferrocyanide and 10 mM EDTA. For *pVPY:GUS*, after GUS staining the roots were fixed in 2.5% glutaraldehyde for 1 h before staining in 0.8 mg mL^−1^ Magenta-GAL (Melford) solution with 100 mM sodium phosphate (pH 7.4), 10 mM KCl and 1 mM MgSO_4_ at 28 °C and in dark overnight. Nodules or root segments were embedded and sections were made as described above. Images were obtained using a digital camera mounted on a Nikon Eclipse E800 microscope.

### Yeast two-hybrid

The constructs for Y2H were made by Gateway cloning. The CDSs of *VPY* and *LIN* were amplified from nodulated Medicago R108 root cDNA by using Phusion High Fidelity Polymerase (NEB) and cloned into pENTR/D-TOPO (Thermo Fisher) or pDONR207 by using Gateway BP Clonase (Invitrogen) to make *pDONR-VPY* and *pDONR-LIN* respectively. The different fragments of *VPY* or *LIN* were then amplified from *pDONR-VPY* or *-LIN*, ligated into pDONR-207 to make pDONR-MSP, -ankyrin, -L1, -L2, -L3, -L4, -L5 constructs*. pDONR*-*VPYL* and *pDONR*-*LINL* were made in the same way except that they were synthesized (Life Technologies). These constructs were then cloned into destination vector pDEST-GBKT7 or pDEST-GADT7 by using Gateway LR Clonase (Invitrogen) to make yeast BD or AD constructs. AD-VPY, -MSP, -ankyrin, -VPYL, -LINL were transformed into yeast strain AH109. BD-LIN, -L1, -L2, -L3, -L4, -L5, -VPY, -VPYL were transformed into yeast strain Y187. The procedure for yeast transformation followed the PEG/LiAC protocol (Clontech). Yeast mating experiment and drop tests was used to check protein-protein interactions (Clontech). For mating experiments, yeast transformed with empty pDEST-GBKT7 (BD-) or pDEST-GADT7 (AD-) were used as negative controls and BD-p53 + AD-T was used as a positive control. Yeast was allowed to grow 3 days on SD-Leu-Trp (growth control) and SD-Ade-His-Leu-Trp before pictures were taken.

### In vivo imaging of fluorescent protein fusions in root hairs

A construct containing *pVPY:VPY-GFP* and *pAtUBQ10:DsRED* was used to study VPY subcellular localization. The CaMV 35 S promoter was removed from Gateway vector pK7FWG2-R by *Spe*I, *Hind*III double digestion, blunting and re-ligation to make pK7FWG2-RSH. A fragment containing *pVPY-VPY* was amplified from *pVPY:VPY-GFP* (pCAMBIA2301)^43^ then ligated into a gateway entry vector before insertion into pK7FWG2-RSH. Due to leaky expression of the GFP in *A. rhizogenes* which interfered with imaging a new version of the construct was made in which *GFP* was replaced by a potato ST-LS1 intron^79^-containing version of *GFP* using the *Eco*RV and *Sac*II restriction sites. *pLjUBQ1:GFP-MSP* and *pLjUBQ1:GFP-ankyrin* were made by Gateway cloning using *pDONR-MSP, pDONR-ankyrin* and a modified gateway destination vector pK7WGF2-R (in which the CaMV 35S promoter was replaced by the *LjUBQ1* promoter). *pLjUBQ1:GFP-LIN*, *pLjUBQ1:GFP-VPYL* and *pLjUBQ1:GFP-EXO70H4* were made in the same way. A confocal laser microscopy live cell imaging system was used in this study^30^. Two to three weeks after hairy root transformation, composite plants transformed with fluorescence fusion constructs were transferred to square petri dishes containing modified Fåhraeus medium with 3 mM MgSO4, 0.5% Phytagel (Sigma) and 50 or 100 nM 2-amino ethoxyvinyl glycine (AVG). The roots were covered with Lumox film (Sarstedt, UK). Plants were then grown vertically in growth chamber at 25 °C with 16 h/8 h light/dark photoperiod and light intensity of 70 μE s^−1^ m^−2^. More than 30 composite A17 plants unless otherwise mentioned were inoculated with Rm1021-CFP or Sm2011-CFP (OD600 = 0.001) and from 2 dpi onwards root hairs at different infection stages were imaged under a Leica SP2 or SP5 confocal laser scanning microscope or spinning disk confocal system. The excitation wavelengths for CFP, GFP and DsRED or autofluorescence of root hair walls were 457, 488 and 561 nm respectively. Fluorescent signals were collected at 465–485 nm (CFP), 500–530 nm (GFP), 570–610 nm (DsRED) or 620–720 nm (autofluorescence). Pseudo-colours presented in figures for CFP, GFP, and DsRED or autofluorescence were magenta, green and red respectively. Confocal images were processed in Leica AF Lite and Fiji^80^ (https://fiji.sc/) to give maximal z-projections of stacks and merged pictures.

The constructs for co-localization of VPY with LIN, LINL and EXO70H4 were made by Golden gate cloning^78^. DNA fragments of *VPY, LIN, LINL, EXO70H4* and a 2.7 kb promoter of *VPY* (*pVPY*) were synthesized by Life Technologies and used as level 0 modules. Level 1 vectors were then assembled to make *pVPY:VPY-GFP*, *pLjUBQ1:mCherry-LIN, pLjUBQ1:mCherry-LINL* and *pLjUBQ1:mCherry-EXO70H4*. Level 1 vectors were then assembled into a level 2 vector EC50505 to make *pVPY:VPY-GFP pLjUBQ1:mCherry-LIN*, *pVPY:VPY-GFP pLjUBQ1:mCherry-LINL* and *pVPY:VPY-GFP pLjUBQ1:mCherry-EXO70H4* respectively. At least 50 root hairs from 10 composite plants containing the different constructs were live cell imaged at 7 dpi with Rm1021 or Rm1021-CFP. under a Leica SP5 confocal laser scanning microscope. The excitation wavelengths for GFP and mCherry were 457 and 561 nm, respectively and signals were collected at 500–530 nm (GFP) and 600–630 nm (mCherry) and pseudo coloured in green and red, respectively.

BiFC constructs were made using Golden Gate cloning. *pVPY*, *VPY*, MSP domain (*MSP*), Ankyrin repeats (*ankyrin*), *LIN*, *EXO70H4* and the split VENUS sequences C-Venus and N-Venus^50^ were synthesized and used as level 0. Level1 constructs were then assembled to make *pVPY:VPY-C-Venus*, *pVPY:MSP-C-Venus*, *pVPY:ankyrin-C-Venus*, *pLjUBQ1:N-Venus-LIN*, *pLjUBQ1:N-Venus-EXO70H4*. Then level 1 constructs were assembled into EC50505, adding *pAtUBQ10:DsRED* as a transgenic marker to make *pVPY:VPY-C-Venus pLjUBQ1:N-Venus-LIN pAtUBQ10:DsRED*, *pVPY:MSP-C-Venus pLjUBQ1:N-Venus-LIN pAtUBQ10:DsRED*, *pVPY:ankyrin-C-Venus pLjUBQ1:N-Venus-LIN pAtUBQ10:DsRED*, *pVPY:VPY-C-Venus pLjUBQ1:N-Venus-EXO70H4 pAtUBQ10:DsRED*, *pVPY:MSP-C-Venus pLjUBQ1:N-Venus-EXO70H4 pAtUBQ10:DsRED* and *pVPY:ankyrin-C-Venus pLjUBQ1:N-Venus-EXO70H4 pAtUBQ10:DsRED*. At least 50 root hairs from 10 DsRED positive composite plants unless otherwise mentioned were selected for analysis by live cell imaging^30^ under a Leica SP5 confocal laser scanning microscope 3 dpi, 4 dpi or 7 dpi with either Rm1021-CFP or Sm2011-CFP (OD600 = 0.001). The excitation wavelengths for Venus and DsRED were 514 nm and 561 nm respectively and signals were collected at 520–545 nm (Venus) and 570–610 nm (DsRED) and pseudo coloured yellow and red, respectively.

### Complementation

For complementation of *vpy*, the construct *pVPY:VPY-GFP pAtUBQ10:DsRED* was introduced into the *vpy-2* mutant by *A. rhizogenes*-mediated hairy root transformation. For complementation of *lin-4* and *lin-1*, *pLIN:GFP-LIN pAtUBQ10:DsRED* and *pLjUBQ1:GFP-LIN pAtUBQ10:DsRED* were introduced by *A. rhizogenes*-mediated hairy root transformation, respectively. 3-week-old composite plants were transferred to sand:terra green and were inoculated with Rm1021 and grown in a controlled environment chamber as described above. At least 30 composite plants were phenotyped at 28 dpi or 60 dpi, unless mentioned otherwise.

### Quantification of GFP fluorescence

The construct *pVPY:VPY-GFP pAtUBQ10:DsRED* was introduced into WT (A17) and *lin-1* mutant composite plants. DsRED positive transgenic plants were inoculated with Rm1021 (OD600 = 0.001) and root hairs were imaged at 7 dpi using a Leica SP8 confocal laser microscope. All parameters were the same for WT/*pVPY:VPY-GFP pAtUBQ10:DsRED* and *lin-1*/*pVPY:VPY-GFP pAtUBQ10:DsRED* plants and only root hairs without any deformation were used for quantification. The number of puncta of VPY-GFP from each root hair were counted and the average number of puncta per root hair was compared between WT and *lin-1* transgenic plants. The intensities of the brightest punctus in each root hair were quantified in Fiji^80^ (https://fiji.sc/). Student’s *t-* test was applied for statistical analysis.

### Transmission electron microscopy

Wild type (R108), *vpy-1* and *vpy-2* seedlings were grown in sand:terra green and inoculated with Rm1021. Nodules were harvested at 47 dpi for Transmission Electron Microscropy. The nodules were cut in half lengthways and immediately placed in a solution of 2.5% (v/v) glutaraldehyde in 0.05 M sodium cacodylate, pH 7.3 for fixation, and left overnight at room temperature. Samples were then placed in baskets and loaded into the Leica EM TP embedding machine (Leica, Milton Keynes) using the following protocol. The fixative was washed out by three successive 15-minute washes in 0.05 M sodium cacodylate and post-fixed in 1% (w/v) OsO_4_ in 0.05 M sodium cacodylate for two hours at room temperature. The osmium fixation was followed by three, 15-minute washes in distilled water before beginning the ethanol dehydration series (30, 50, 70, 95 and two changes of 100% ethanol, each for an hour). Once dehydrated, the samples were gradually infiltrated with LR White resin (London Resin Company, Reading, Berkshire) by successive changes of resin:ethanol mixes at room temperature (1:1 for 1 h, 2:1 for 1 h, 3:1 for 1 h, 100% resin for 1 h then 100% resin for 16 h and a fresh change again for a further 8 h) then the samples were transferred into gelatin capsules full of fresh LR White and placed at 60 ^o^C for 16 h to polymerize. The material was sectioned with a diamond knife using a Leica UC6 ultramicrotome (Leica, Milton Keynes) and ultrathin sections of approximately 90 nm were picked up on 200 mesh copper grids which had been pyroxylin and carbon coated. The sections were stained with 2% (w/v) uranyl acetate for 1 h and 1% (w/v) lead citrate for 1 min, washed in distilled water and air dried. The grids were viewed in a FEI Tecnai 20 transmission electron microscope (FEI UK Ltd, Cambridge, UK) at 200 kV and imaged using an AMT XR60 digital camera (Deben, Bury St Edmunds, UK) to record TIF files.

### Reporting summary

Further information on research design is available in the Nature Research Reporting Summary linked to this article.

## Supplementary information

Supplementary Information

Description of Additional Supplementary Files

Supplementary Movie 1

Supplementary Movie 2

Reporting Summary

Source Data

## Data Availability

The authors declare that all data supporting the findings of this study are available within the article and its Supplementary Information Files. The source data underlying Figs. 7b, 7c, 10a, 10c and Supplementary Figs. 3, 15, 18, 19 are provided in the source data file. Materials generated in this study are available from the corresponding authors upon reasonable request.

## References

[1] Downie JA (2014). Legume nodulation. Curr. Biol..

[2] Xiao TT (2014). Fate map of *Medicago truncatula* root nodules. Development.

[3] Oldroyd GE, Murray JD, Poole PS, Downie JA (2011). The rules of engagement in the legume-rhizobial symbiosis. Annu. Rev. Genet..

[4] Madsen LH (2010). The molecular network governing nodule organogenesis and infection in the model legume *Lotus japonicus*. Nat. Commun..

[5] Oldroyd GE, Downie JA (2008). Coordinating nodule morphogenesis with rhizobial infection in legumes. Annu. Rev. Plant Biol..

[6] Broghammer A (2012). Legume receptors perceive the rhizobial lipochitin oligosaccharide signal molecules by direct binding. Proc. Natl Acad. Sci. USA.

[7] Kelly S, Radutoiu S, Stougaard J (2017). Legume LysM receptors mediate symbiotic and pathogenic signalling. Curr. Opin. Plant Biol..

[8] Ehrhardt DW, Wais R, Long SR (1996). Calcium spiking in plant root hairs responding to *Rhizobium* nodulation signals. Cell.

[9] Charpentier (2008). *Lotus japonicus* CASTOR and POLLUX are ion channels essential for perinuclear calcium spiking in legume root endosymbiosis. Plant Cell.

[10] Charpentier M (2016). Nuclear-localized cyclic nucleotide-gated channels mediate symbiotic calcium oscillations. Science.

[11] Lévy J (2004). A putative Ca^2+^ and calmodulin-dependent protein kinase required for bacterial and fungal symbioses. Science.

[12] Mitra RM (2004). A Ca^2+^/calmodulin-dependent protein kinase required for symbiotic nodule development: Gene identification by transcript-based cloning. Proc. Natl Acad. Sci. USA.

[13] Barker DG, Chabaud M, Russo G, Genre A (2017). Nuclear Ca^2+^ signalling in arbuscular mycorrhizal and actinorhizal endosymbioses: on the trail of novel underground signals. New Phytol..

[14] Parniske M (2008). Arbuscular mycorrhiza: the mother of plant root endosymbioses. Nat. Rev. Microbiol..

[15] Yano K (2008). CYCLOPS, a mediator of symbiotic intracellular accommodation. Proc. Natl Acad. Sci. USA.

[16] Schauser L, Roussis A, Stiller J, Stougaard J (1999). A plant regulator controlling development of symbiotic root nodules. Nature.

[17] Smit P (2005). NSP1 of the GRAS protein family is essential for rhizobial Nod factor-induced transcription. Science.

[18] Soyano T, Kouchi H, Hirota A, Hayashi M (2013). Nodule inception directly targets NF-Y subunit genes to regulate essential processes of root nodule development in *Lotus japonicus*. PLoS Genet..

[19] Andriankaja A (2007). AP2-ERF transcription factors mediate Nod factor dependent *MtENOD11* activation in root hairs via a novel cis-regulatory motif. Plant Cell.

[20] Middleton PH (2007). An ERF transcription factor in *Medicago truncatula* that is essential for Nod factor signal transduction. Plant Cell.

[21] Kalo P (2005). Nodulation signaling in legumes requires NSP2, a member of the GRAS family of transcriptional regulators. Science.

[22] Marsh JF (2007). *Medicago truncatula* NIN is essential for rhizobial independent nodule organogenesis induced by autoactive calcium/calmodulindependent protein kinase. Plant Physiol..

[23] Laloum T (2014). Two CCAATbox-binding transcription factors redundantly regulate early steps of the legume-rhizobia endosymbiosis. Plant J..

[24] Laporte P (2014). The CCAAT box-binding transcription factor NF-YA1 controls rhizobial infection. J. Exp. Bot..

[25] Cerri MR (2017). The ERN1 transcription factor gene is a target of the CCaMK/CYCLOPS complex and controls rhizobial infection in *Lotus japonicus*. New Phytol..

[26] Kawaharada Y (2017). The ethylene responsive factor required for nodulation 1 (ERN1) transcription factor is required for infection-thread formation in *Lotus japonicus*. Mol. Plant Microbe Interact..

[27] Gage DJ (2004). Infection and invasion of roots by symbiotic, nitrogenfixing rhizobia during nodulation of temperate legumes. Microbiol. Mol. Biol. Rev..

[28] Murray JD (2011). Invasion by invitation: rhizobial infection in legumes. Mol. Plant Microbe Interact..

[29] Fournier J (2015). Remodeling of the infection chamber before infection thread formation reveals a two-step mechanism for rhizobial entry into the host legume root hair. Plant Physiol..

[30] Fournier J (2008). Mechanism of infection thread elongation in root hairs of *Medicago truncatula* and dynamic interplay with associated rhizobial colonization. Plant Physiol..

[31] Ivanov S (2012). Rhizobium-legume symbiosis shares an exocytotic pathway required for arbuscule formation. Proc. Natl. Acad. Sci. U. S. A..

[32] Xie F (2012). Legume pectate lyase required for root infection by rhizobia. Proc. Natl Acad. Sci. USA.

[33] Liu Cheng-Wu, Breakspear Andrew, Guan Dian, Cerri Marion R., Jackson Kirsty, Jiang Suyu, Robson Fran, Radhakrishnan Guru V., Roy Sonali, Bone Caitlin, Stacey Nicola, Rogers Christian, Trick Martin, Niebel Andreas, Oldroyd Giles E.D., de Carvalho-Niebel Fernanda, Murray Jeremy D. (2019). NIN Acts as a Network Hub Controlling a Growth Module Required for Rhizobial Infection. Plant Physiology.

[34] Arrighi JF (2008). The *RPG* gene of *Medicago truncatula* controls Rhizobium-directed polar growth during infection. Proc. Natl Acad. Sci. USA.

[35] Hossain MS (2012). *Lotus japonicus* ARPC1 is required for rhizobial infection. Plant Physiol..

[36] Yokota K (2009). Rearrangement of actin cytoskeleton mediates invasion of *Lotus japonicus* roots by *Mesorhizobium loti*. Plant Cell.

[37] Miyahara A (2010). Conservation in function of a SCAR/WAVE component during infection thread and root hair growth in *Medicago truncatula*. Mol. Plant Microbe Interact..

[38] Qiu L (2015). SCARN a novel class of SCAR protein that is required for root hair infection during legume nodulation. PLoS Genet..

[39] Sinharoy S (2016). A *Medicago truncatula* cystathionine-β-synthase-like domain-containing protein is required for rhizobial infection and symbiotic nitrogen fixation. Plant Physiol..

[40] Kiss E (2009). LIN, a novel type of U-box/WD40 protein, controls early infection by rhizobia in legumes. Plant Physiol..

[41] Yano K (2009). CERBERUS, a novel U-box protein containing WD-40 repeats, is required for formation of the infection thread and nodule development in the legume-Rhizobium symbiosis. Plant J..

[42] Murray JD (2011). *Vapyrin*, a gene essential for intracellular progression of arbuscular mycorrhizal symbiosis, is also essential for infection by rhizobia in the nodule symbiosis of *Medicago truncatula*. Plant J..

[43] Pumplin N (2010). *Medicago truncatula* Vapyrin is a novel protein required for arbuscular mycorrhizal symbiosis. Plant J..

[44] Feddermann N (2010). The *PAM1* gene of petunia, required for intracellular accommodation and morphogenesis of arbuscular mycorrhizal fungi, encodes a homologue of VAPYRIN. Plant J..

[45] Feddermann N, Reinhardt D (2011). Conserved residues in the ankyrin domain of VAPYRIN indicate potential protein-protein interaction surfaces. Plant Signal. Behav..

[46] Zhang X, Pumplin N, Ivanov S, Harrison MJ (2015). EXO70I is required for development of a sub-domain of the periarbuscular membrane during arbuscular mycorrhizal symbiosis. Curr. Biol..

[47] Guan D (2013). Rhizobial infection is associated with the development of peripheral vasculature in nodules of *Medicago truncatula*. Plant Physiol..

[48] Breakspear A (2014). The root hair “infectome” of *Medicago truncatula* uncovers changes in cell cycle genes and reveals a requirement for Auxin signaling in rhizobial infection. Plant Cell.

[49] Kuppusamy KT (2004). *LIN*, a *Medicago truncatula* gene required for nodule differentiation and persistence of rhizobial infections. Plant Physiol..

[50] Gehl C (2009). New GATEWAY vectors for high throughput analyses of protein-protein interactions by bimolecular fluorescence complementation. Mol. Plant.

[51] Komander D, Rape M (2012). The ubiquitin code. Annu. Rev. Biochem..

[52] Tadege M (2008). Large-scale insertional mutagenesis using the *Tnt1* retrotransposon in the model legume *Medicago truncatula*. Plant J..

[53] Oldroyd GE (2013). Speak, friend, and enter: signalling systems that promote beneficial symbiotic associations in plants. Nat. Rev. Microbiol..

[54] He B, Guo W (2009). The exocyst complex in polarized exocytosis. Curr. Opin. Cell Biol..

[55] Martin-Urdiroz, M., Deeks, M. J., Horton, C. G., Dawe, H. R. & Jourdain, I. The exocyst complex in health and disease. *Front*. *Cell**Dev*. *Biol*. **4**, 24, 10.3389/fcell.2016.00024 (2016). 10.3389/fcell.2016.00024 PMC4828438 27148529

[56] Takeda N (2013). CERBERUS and NSP1 of *Lotus japonicus* are common symbiosis genes that modulate arbuscular mycorrhiza development. Plant Cell Physiol..

[57] Nagae M, Takeda N, Kawaguchi M (2014). Common symbiosis genes CERBERUS and NSP1 provide additional insight into the establishment of arbuscular mycorrhizal and root nodule symbioses in *Lotus japonicus*. Plant Signal. Behav..

[58] Bonfante P, Genre A (2010). Mechanisms underlying beneficial plant-fungus interactions in mycorrhizal symbiosis. Nat. Commun..

[59] Martin FM, Uroz S, Barker DG (2017). Ancestral alliances: Plant mutualistic symbioses with fungi and bacteria. Science.

[60] Genre A (2005). Arbuscular mycorrhizal fungi elicit a novel intracellular apparatus in *Medicago truncatula* root epidermal cells before infection. Plant Cell.

[61] Gutjahr C, Parniske M (2013). Cell and developmental biology of arbuscular mycorrhiza symbiosis. Annu. Rev. Cell Dev. Biol..

[62] Zhang Y, Liu CM, Emons AM, Ketelaar T (2010). The plant exocyst. J. Integr. Plant Biol..

[63] Li S (2010). Expression and functional analyses of EXO70 genes in Arabidopsis implicate their roles in regulating cell type-specific exocytosis. Plant Physiol..

[64] Cvrcková F (2012). Evolution of the land plant exocyst complexes. Front. Plant Sci..

[65] Zarsky V, Kulich I, Fendrych M, Pecenkova T (2013). Exocyst complexes multiple functions in plant cells secretory pathways. Curr. Opin. Plant Biol..

[66] Harrison MJ, Ivanov S (2017). Exocytosis for endosymbiosis: membrane trafficking pathways for development of symbiotic membrane compartments. Curr. Opin. Plant Biol..

[67] Pecenková T (2011). The role for the exocyst complex subunits Exo70B2 and Exo70H1 in the plant-pathogen interaction. J. Exp. Bot..

[68] Kulich I (2015). Cell wall maturation of Arabidopsis trichomes is dependent on exocyst subunit EXO70H4 and involves callose deposition. Plant Physiol..

[69] Gavrin A, Kulikova O, Bisseling T, Fedorova EE (2017). Interface symbiotic membrane formation in root nodules of *Medicago truncatula*: the role of synaptotagmins *MtSyt1*, *MtSyt2* and *MtSyt3*. Front. Plant Sci..

[70] Sekereš J (2017). Analysis of exocyst subunit EXO70 family reveals distinct membrane domains in tobacco pollen tubes. Plant Physiol..

[71] Genre A (2012). Multiple exocytotic markers accumulate at the sites of perifungal membrane biogenesis in arbuscular mycorrhizas. Plant Cell Physiol..

[72] Boyd C, Hughes T, Pypaert M, Novick P (2004). Vesicles carry most exocyst subunits to exocytic sites marked by the remaining two subunits, Sec3p and Exo70p. J. Cell Biol..

[73] Hane JK (2016). A comprehensive draft genome sequence for lupin (*Lupinus angustifolius*), an emerging health food: insights into plant-microbe interactions and legume evolution. Plant Biotechnol. J..

[74] Sagan M, Morandi D, Tarenghi E, Duc G (1995). Selection of nodulation and mycorrhizal mutants in the model plant *Medicago truncatula* (Gaertn.) after γ -ray mutagenesis. Plant Sci..

[75] Schnabel E, Journet EP, de Carvalho-Niebel F, Duc G, Frugoli J (2005). The *Medicago truncatula SUNN* gene encodes a CLV1-like leucine-rich repeat receptor kinase that regulates nodule number and root length. Plant Mol. Biol..

[76] Cheng HP, Walker GC (1998). Succinoglycan is required for initiation and elongation of infection threads during nodulation of alfalfa by *Rhizobium meliloti*. J. Bacteriol..

[77] Boisson-Dernier A (2001). *Agrobacterium rhizogenes*-transformed roots of *Medicago truncatula* for the study of nitrogen-fixing and endomycorrhizal symbiotic associations. Mol. Plant Microbe Interact..

[78] Patron NJ (2015). Standards for plant synthetic biology: a common syntax for exchange of DNA parts. New Phytol..

[79] Pang S (1996). An improved green-fluorescence protein gene as a vital marker in plants. Plant Physiol..

[80] Schindelin J (2012). Fiji: an open-source platform for biological-image analysis. Nat. methods.

