# Supplementary material for: A protein complex required for polar growth of rhizobial infection threads
Source: Nat Commun. 2019 Jun 28;10:2848. doi: 10.1038/s41467-019-10029-y (PMC6599036; doi:10.1038/s41467-019-10029-y)
Supplement: Supplementary file 1 — Supplementary Information [file 41467_2019_10029_MOESM1_ESM.pdf]

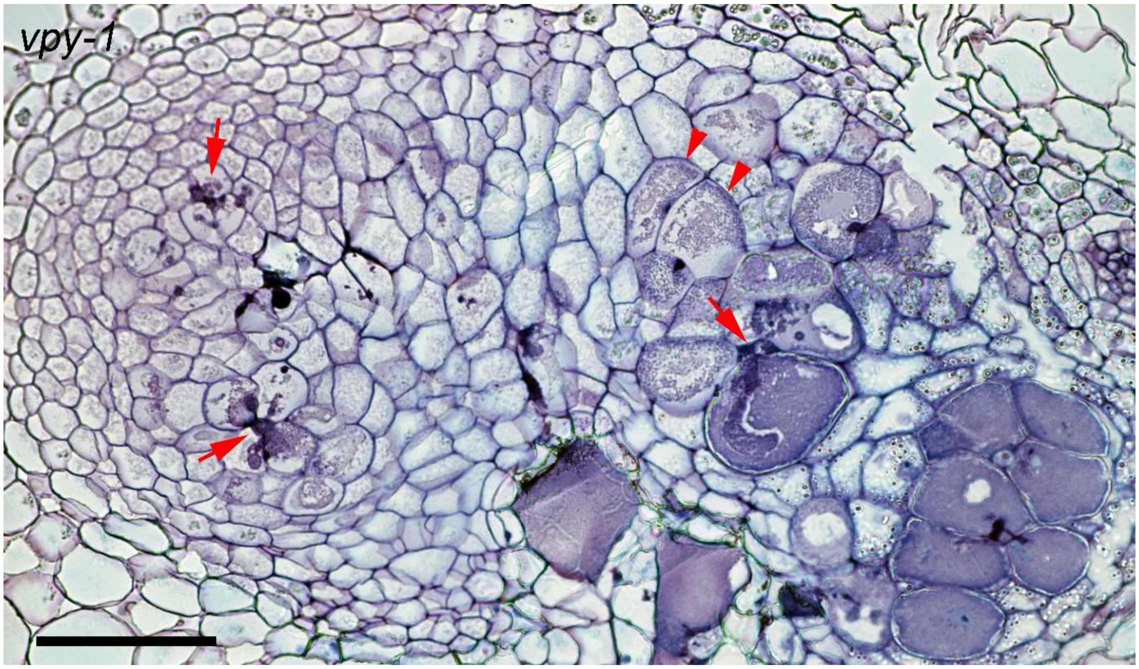

Supplementary Figure 1 Rhizobial infection in *vapyrin-1* nodules. Toluidine blue-stained longitudinal section of *vpy-1* nodule at 47dpi. Arrows indicate the intercellular accumulation of bacteria. Arrowheads indicate cells with low level of rhizobial colonization. Scale bar, 100µm.

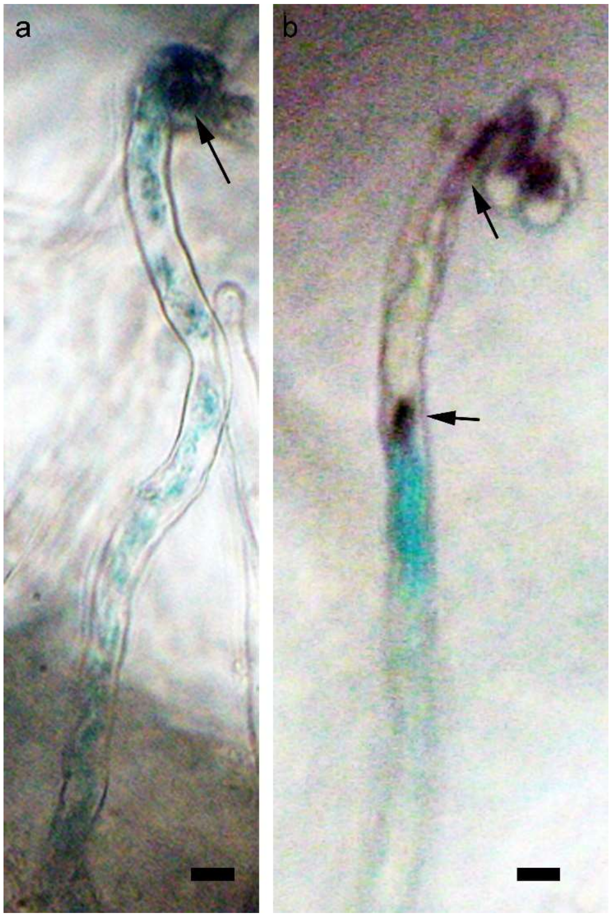

Supplementary Figure 2 Expression of *pVPY:GUS* in root hairs. Images of root hairs in composite plants transformed with *pVPY:GUS* show the expression of *VPY* in root hairs harbouring a microcolony (arrow in **a**) or an infection thread (arrows in **b**). Blue colour indicates GUS staining and magenta colour indicates X-gal staining of *Sinorhizobium meliloti 1021-lacZ*. Scale bar, 10µm.

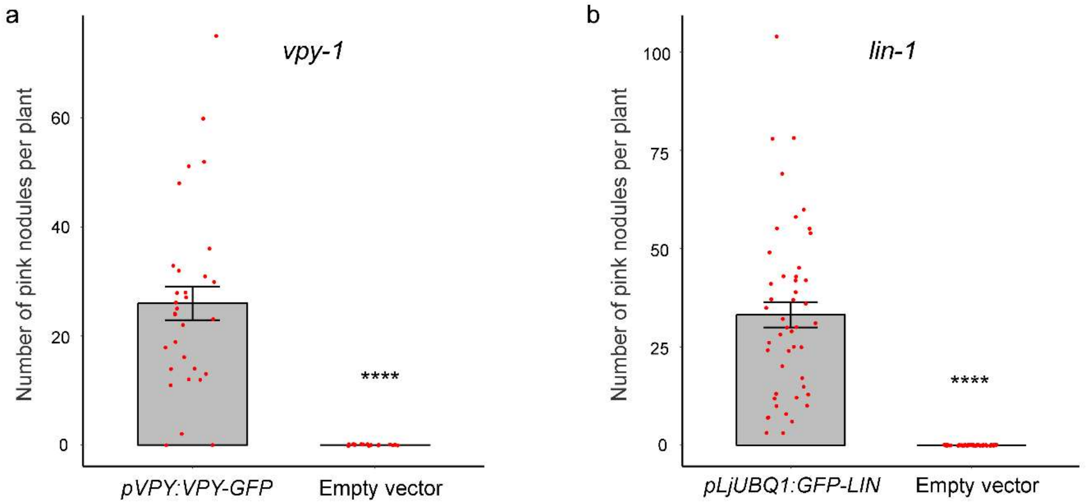

Supplementary Figure 3 Complementation of *vpy-1* and *lumpy infection-1*. Average number of pink nodules per composite transgenic plant of *vpy-1/pVPY:VPY-GFP* or *vpy-1/Empty Vector* (**a**) and *lin-1/pLjUBQ1:GFP-LIN* or *lin-1/Empty vector* (**b**) at 30 days post inoculation with *S.meliloti Rm1021-lacZ*. The proportion of composite plants with pink nodules was 29/31 for *vpy-1/pVPY:VPY-GFP* , 0/20 for *vpy-1/Empty Vector,* 47/47 for *lin-1/pLjUBQ1:GFP-LIN* and 0/57 for *lin-1/Empty vector*. ****, $p<0.0001$, 2-tailed Student's *t*-test. Error bars indicate standard error. Source data are provided as a Source Data file.

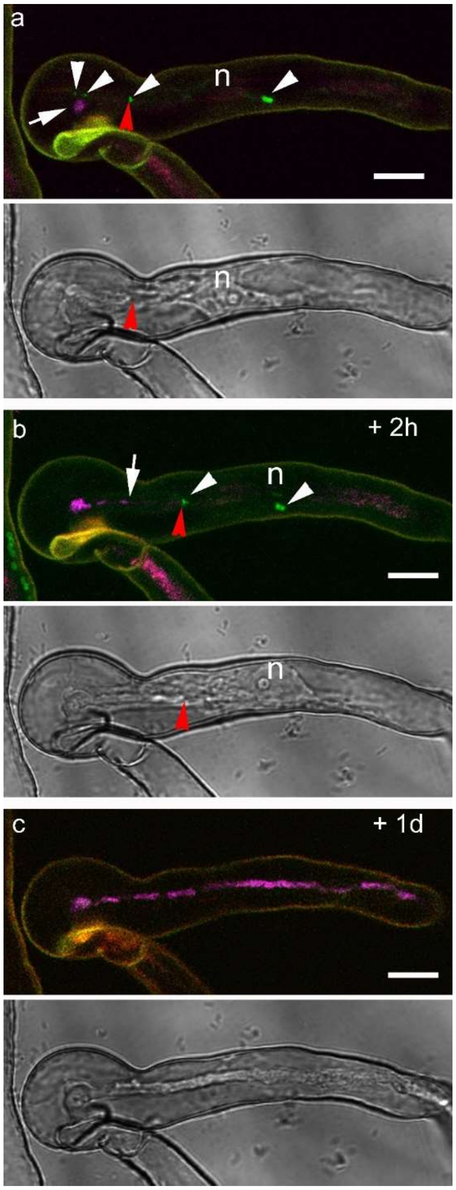

Supplementary Figure 4 Time lapse of VPY-GFP during rhizobial infection. Live cell confocal images showing localization of *pVPY* driving *VPY-GFP* in *sunn-2* root hairs during rhizobial infection. Composite plants transformed with *pVPY:VPY-GFP* were inoculated with *S. meliloti 2011-CFP*. (**a**) The root hair tip was first imaged 2dpi at the beginning of infection thread formation. Note that the rhizobia from the microcolony have not yet propagated towards the tubular infection thread, which tip is associated to a VPY-GFP labeled punctus; (**b**) 2h later, a single file of bacteria is colonizing the elongating infection thread which growing tip is now found about 10 µm lower down the root hair, compared to its position in (a), similarly associated to a VPY-GFP-labeled punctus; and (**c**) 1d later, rhizobial infection is completed, and VPY-GFP punctae absent from the imaged field . Note that DsRed was undetectable in this particular root. Upper images in (**a-c**) are merged ones of cell wall auto-fluorescence + GFP + CFP and lower ones in (**a-c**) are bright field images. n, nucleus. White arrows, CFP tagged rhizobia. White arrowheads, GFP puncta. Red arrowheads, tip of the infection thread. Scale bars, 10 µm. Images are representative of more than 40 ongoing infection sites, monitored in 15 *sunn-2* composite plants in 3 independent experiments.

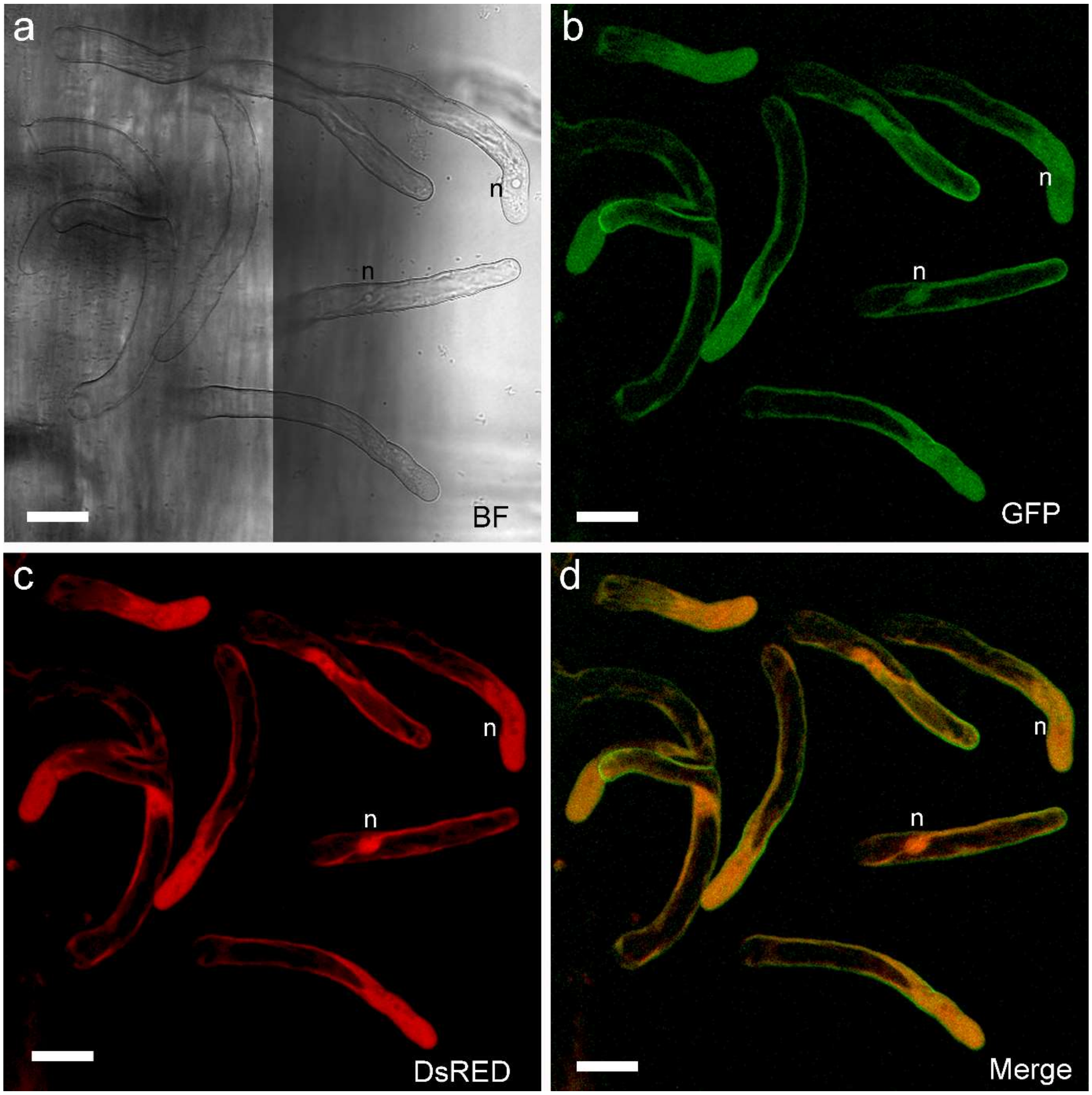

Supplementary Figure 5 Subcellular localization of VPY in non-inoculated root hairs. (**a-d**) Live cell confocal images showing localization of *pVPY* driven *VPY-GFP* in *sunn-2* root hairs in a non-inoculated composite plant (**a**) Bright Field (BF); (**b**) GFP; (**c**) DsRED; (**d**) merged of GFP+DsRED. n, nucleus. Scale bars, 20 µm. 20 growing root hairs were imaged in 7 DsRed-positive roots from 4 plants.

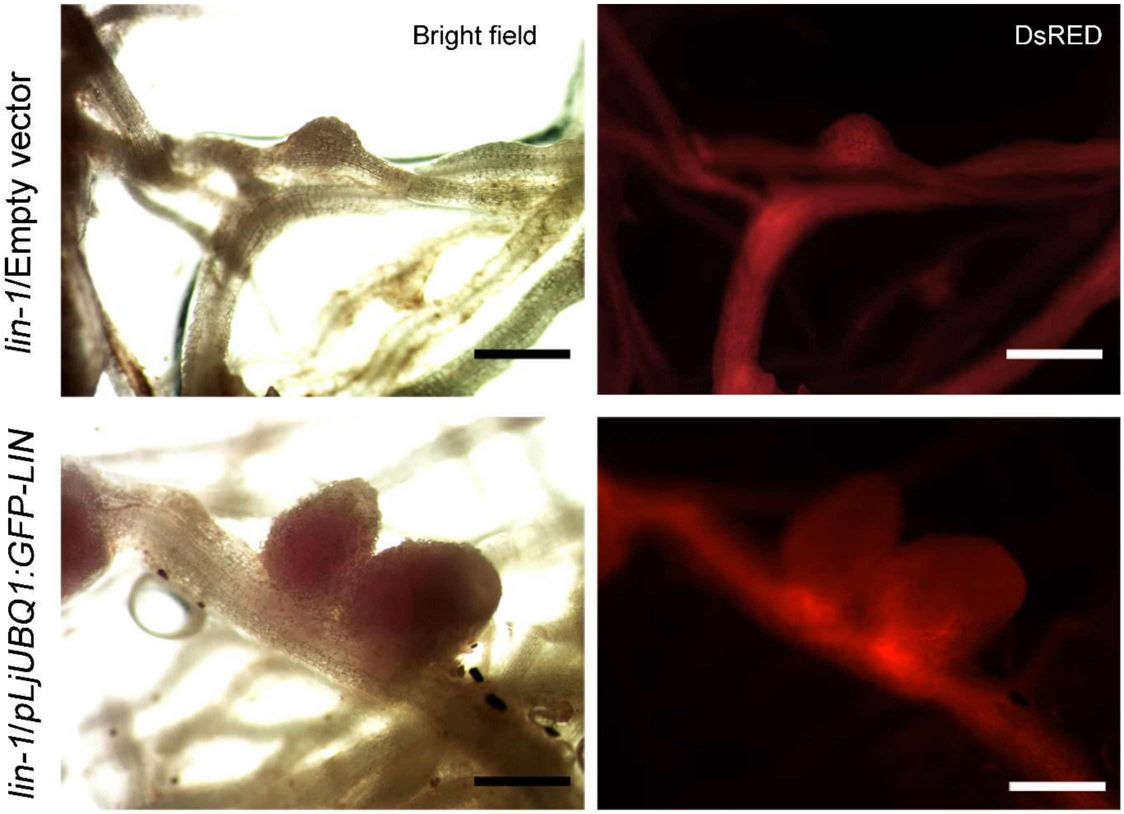

Supplementary Figure 6 Complementation of *lin-1* by *pLjUBQ1:GFP-LIN*. Root segments with nodules of *A. rhizogenes* mediated hairy root *lin-1* plants transformed with either an empty vector (upper) or *pLjUBQ1:GFP-LIN* (bottom) at 4 weeks post inoculation with *S. meliloti*. DsRED was used as a transgenic marker. Scale bars, 1mm.

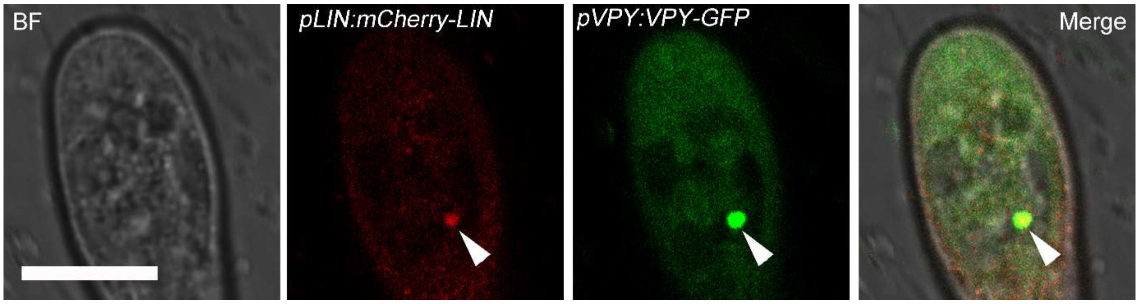

Supplementary Figure 7 Co-localization of VPY and LIN in root hairs. Live cell confocal images from a root hair in a transgenic root which contains *pVPY:VPY-GFP* and *pLIN:mCherry-LIN*, showing co-localization of VPY (green) and LIN (red) after inoculation with *S. meliloti 1021*. BF, bright field. Arrowheads, mcherry/GFP puncta. Scale bar, 10 µm.

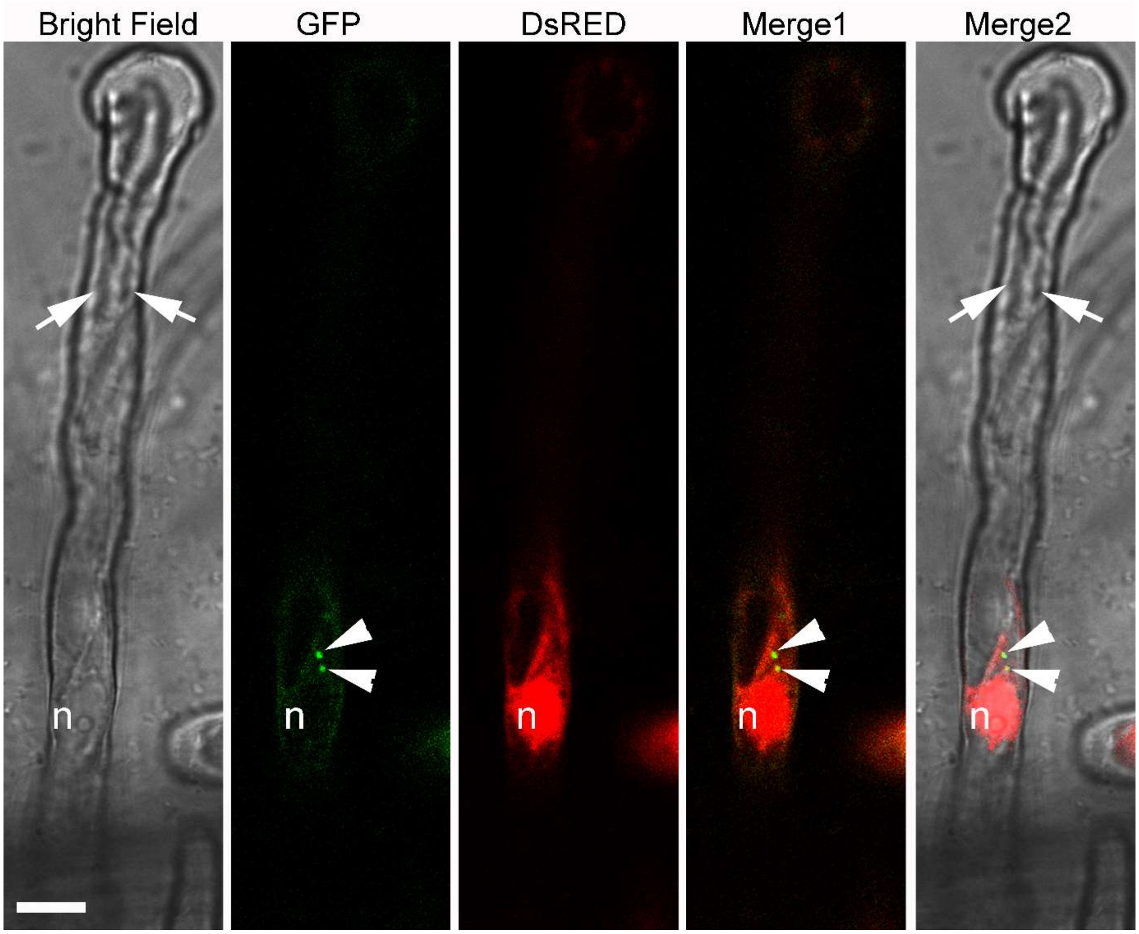

Supplementary Figure 8 Subcellular localization of LIN in root hair harbouring infection threads. Live cell images from a single confocal section showing pLjUBQ1:GFP-LIN puncta (arrowheads) near the nucleus of a root hair which harbours two infection threads (arrows, see the same root hair in Fig. 4t-y). DsRED was used as a transgenic marker. n, nucleus. Scale bar, 10 µm.

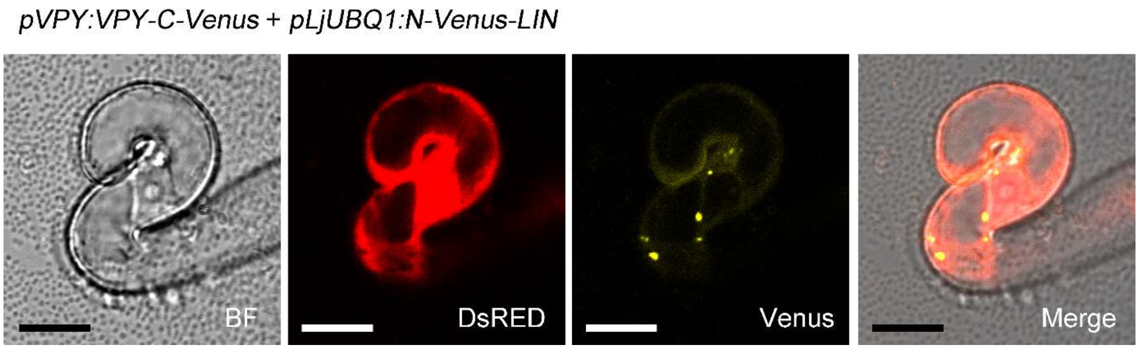

Supplementary Figure 9 Interaction of VPY and LIN shown by Bimolecular Fluorescence Complementation. Confocal images showing a root hair from a composite plant transformed with a construct containing *pVPY:VPY-C-Venus*, *pLjUBQ1:N-Venus-LIN* and *pAtUBQ10:DsRED* as a transgenic marker, 7 dpi with *S. meliloti-1021*. Scale bar, 10µm.

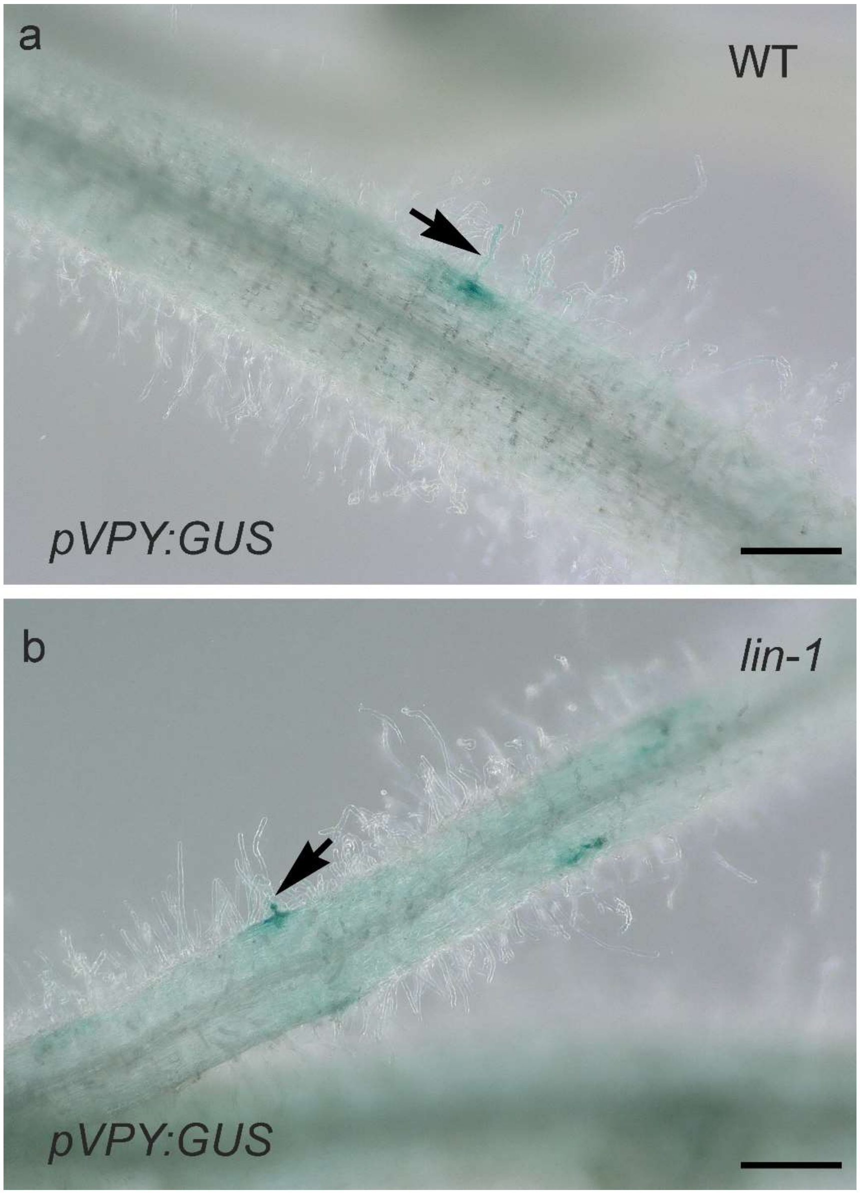

Supplementary Figure 10 Expression of *VPY* in WT and *lin-1*. Images showing GUS activity in *pVPY:GUS* expressing root hairs of WT (**a**) and *lin-1* (**b**) during rhizobial infection. Composite transgenic plants were stained 5 hours for GUS activity at 7 days post inoculation with *S. meliloti* Rm1021-lacZ (n = 14 for WT, 18 for *lin-1*). Blue colour indicates staining of GUS activity and arrows indicate GUS stained root hairs. Scale bars, 100µm.

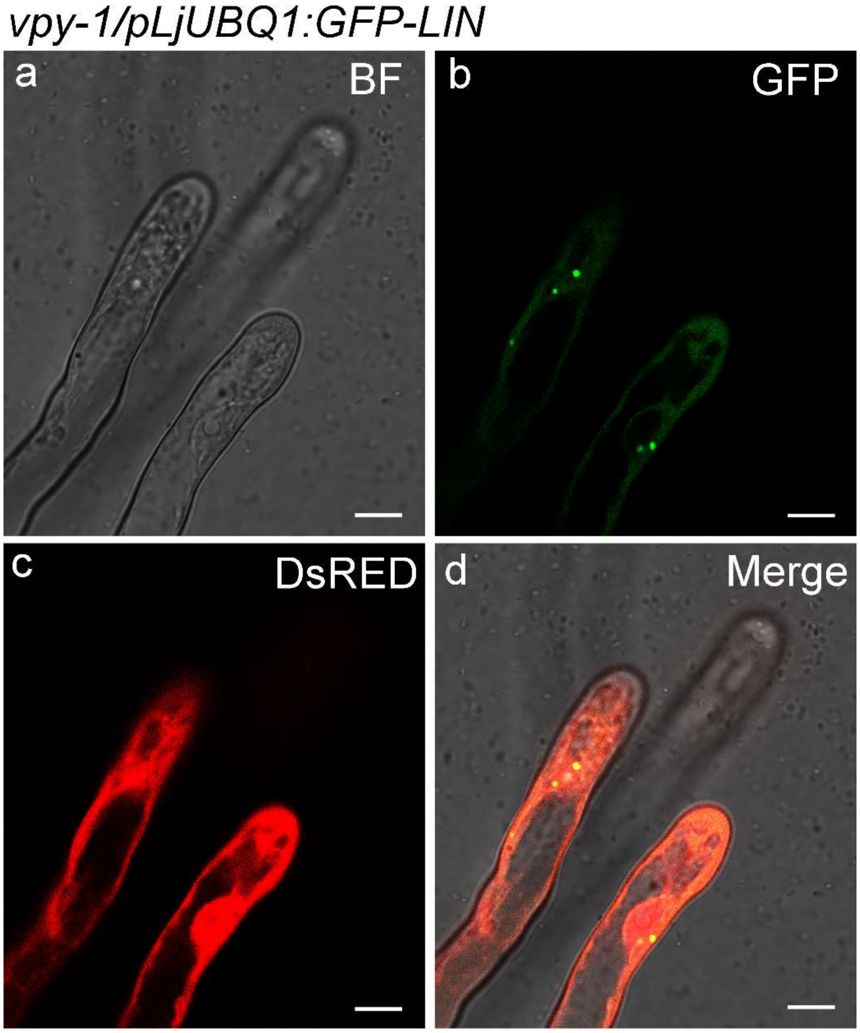

Supplementary Figure 11 Subcellular localization of LIN in *vpy-1* after rhizobial inoculation. Confocal images from composite transgenic plants of GFP-LIN driven by the *LjUBQ1* promoter showing subcellular localization of GFP-LIN in root hairs of *vpy-1* 7 days post inoculation with *S. meliloti* Rm1021-CFP. DsRED was used as a transgenic marker. **a-d** Bright field, GFP, DsRED and merged images. DsRED and GFP were pseudo-coloured in red and green respectively. Scale bars, 10 µm.

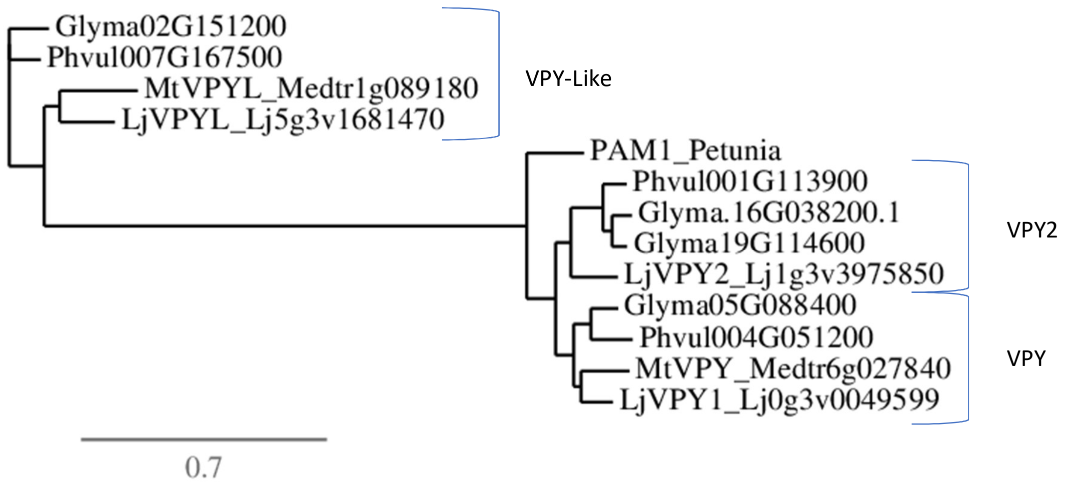

Supplementary Figure 12 A phylogenetic tree of legume VAPYRIN homologs. *Petunia X hybrida* PAM1 is shown for comparison. Maximum likelihood support is indicated for key branches. Bar indicates phylogenetic distance. The tree was created using Phylogeny.fr which employs MUSCLE for amino acid alignments, PhyML for phylogenetic analysis, and TreeDyn for tree rendering[1]. Glyma, *Glycine max*; Mt and Medtr, *Medicago truncatula*; Petunia, *Petunia X hybrida*; Lj, *Lotus japonicus*; Phvul, *Phaseolus vulgaris*. The scale bar indicates the number of substitutions per site.

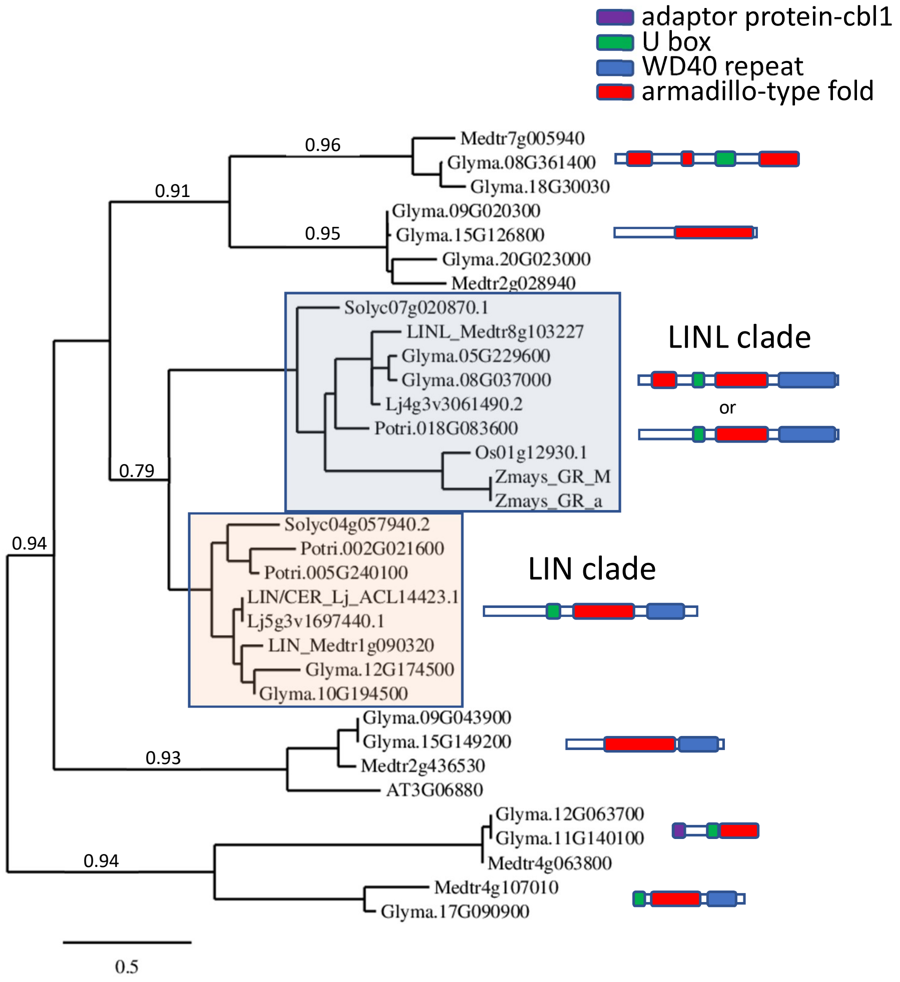

Supplementary Figure 13 A phylogenetic tree of *Glycine max* (Glyma) and *M. truncatula* (Medtr) LIN homologs. *Lotus japonicus* (Lj), *Solanum lycopersicon* (Solyc), *Populus trichocarpa* (Potri), *Oryza sativa* (Os) and *Zea mays* (Zmays) LIN homologs are shown for comparison. The closest Arabidopsis (AT) homolog to LIN is also shown. Maximum likelihood support is indicated for key branches. Bar indicates phylogenetic distance. The tree was created using Phylogeny.fr which employs MUSCLE for amino acid alignments, PhyML for phylogenetic analysis, and TreeDyn for tree rendering[1]. The arrangement of protein domains, as predicted by InterProscan is indicated for each clade. The scale bar indicates the number of substitutions per site.

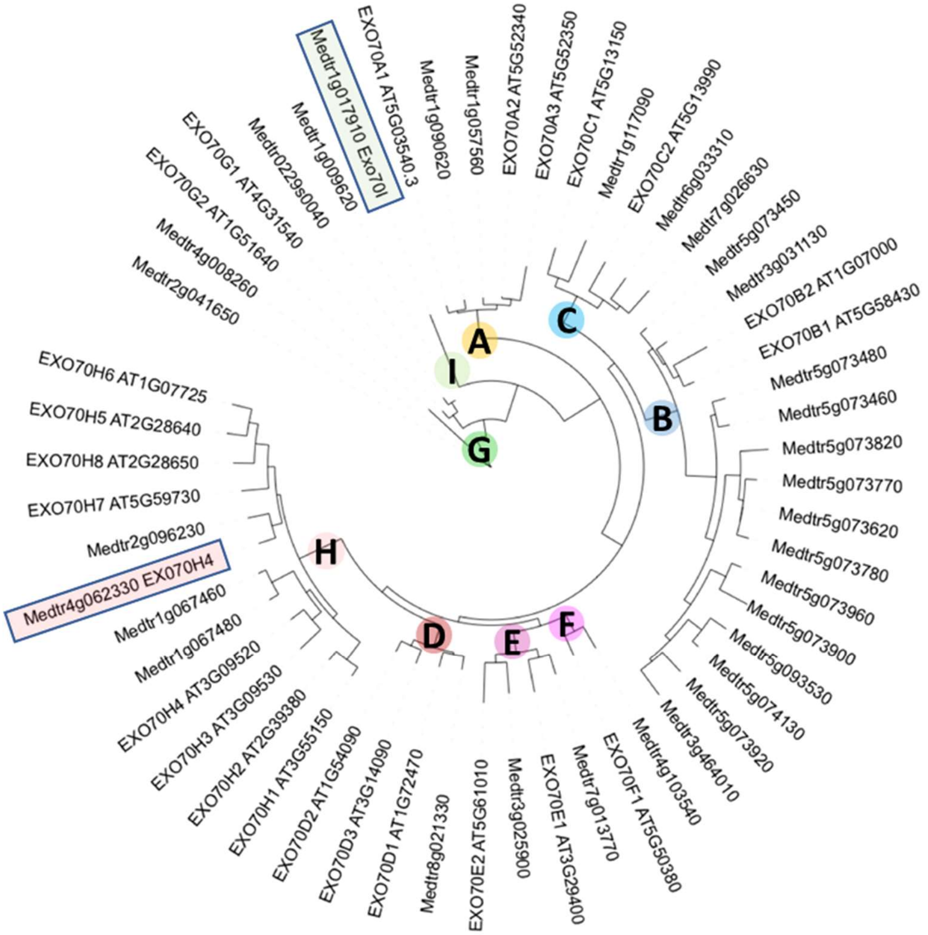

Supplementary Figure 14 A phylogenetic tree of the Arabidopsis and *M. truncatula* (Medtr) EXO70 families. The tree was created using Phylogeny.fr which employs MUSCLE for amino acid alignments, PhyML for phylogenetic analysis. iTOL v1.0 was used for tree rendering[2].

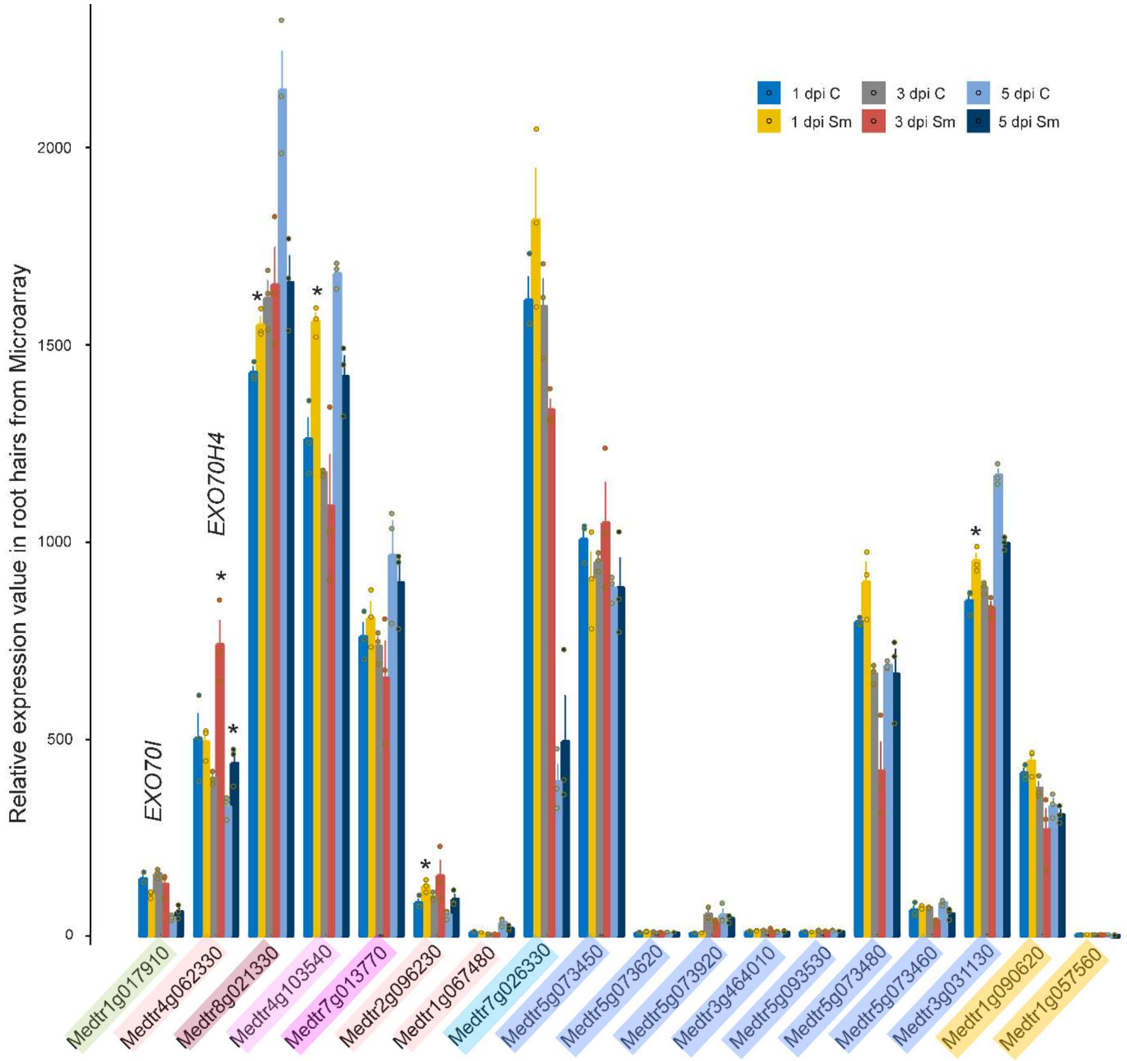

Supplementary Figure 15 Expression of *EXO70* genes in root hairs. Expression of *M. truncatula EXO70s* in root hairs at 1, 3 and 5 days post inoculation with either *S. meliloti nodΔD1ABC* (C) or WT *S. meliloti Rm1021-lacZ* ("Sm"), based on microarray data[3]. Error bars represent standard error. *p<0.05, 2-tailed Student's *t*-test. All *EXO70*s that have microarray probesets are shown with gene models (Mt4.0 v1), using the same colour code as Supplementary Figure 14 to indicate subfamilies. EXO70H4 (Medtr1g017910) is induced 3 and 5 dpi and 4 other *EXO70s* (Medtr8g021330, Medtr4g103540, Medtr2g096230 and Medtr3g031130) are induced at 1dpi. *EXO70I* and *EXO70H4* are labelled in the bar chart. Source data are provided as a Source Data file.

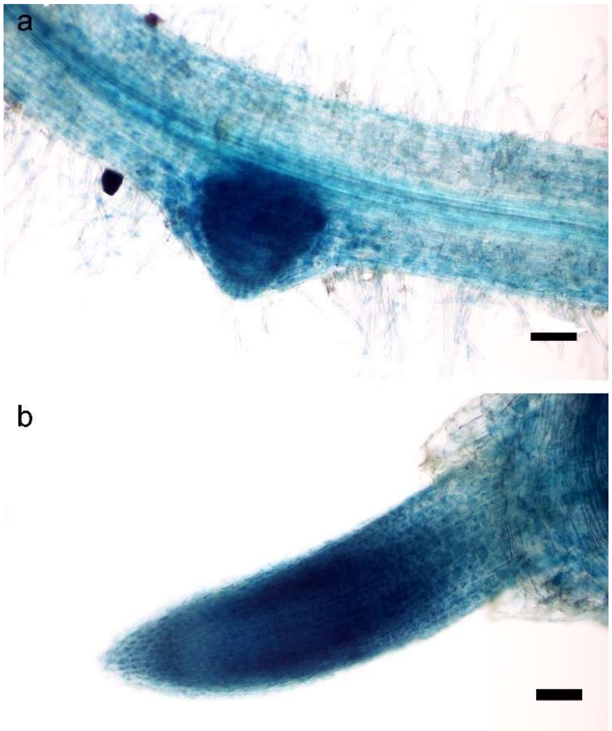

Supplementary Figure 16 Expression of *EXO70H4-GUS* Promoter Fusion in *M. truncatula* roots.
pEXO70H4:GUS expression in composite plants showing expression in a lateral root primordium (**a**) and a lateral root (**b**). Scale bar, 100µm.

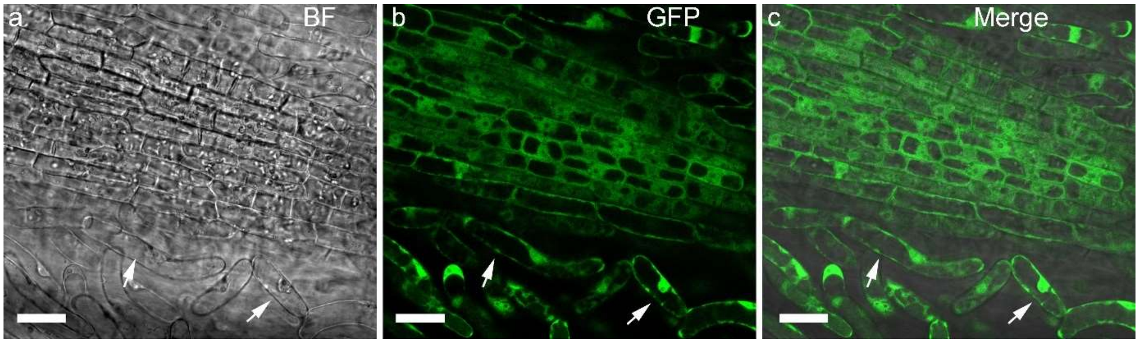

Supplementary Figure 17 Localization of *pLjUBQ1*-driven GFP-EXO70H4 in root tip cells. Confocal images of GFP-EXO70H4 in the cells at the root tip and border cells (arrows) in composite plants (**a-c**). BF, bright field. Scale bar, 20µm.

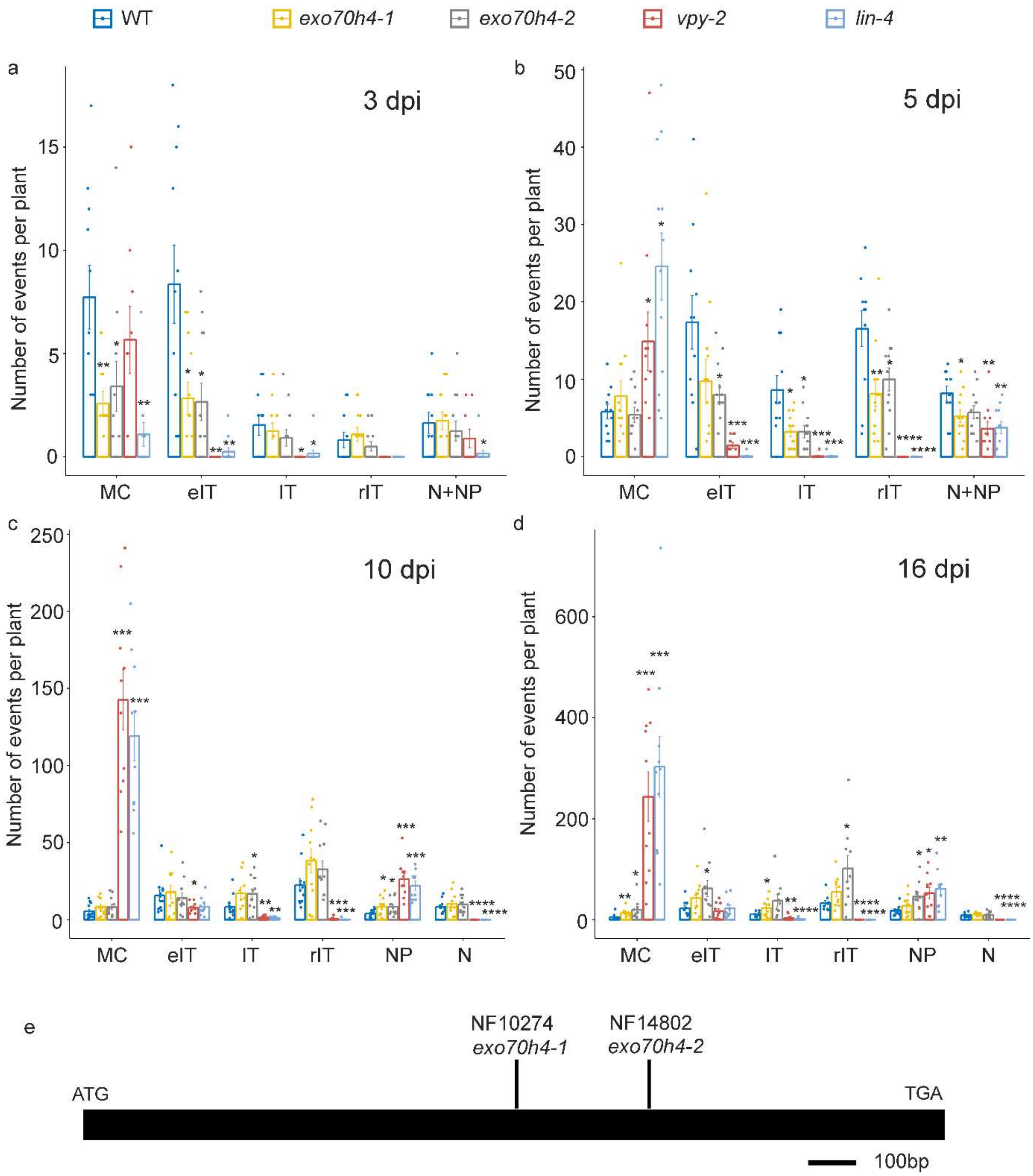

Supplementary Figure 18 Time course quantification of different stages of rhizobial infection events in WT, *exo70h4*, *vpy-2* and *lin-4*. (a-d) All infection events from microcolony to ramified cortical infection threads were counted from 9-12 plants for each genotype at 3, 5, 10 and 16 dpi with *S. meliloti* Rm1021-lacZ respectively. **(e)** *M. truncatula* lines NF10274 and NF14802 containing *Tnt1* insertions at 977bp (*exo70h4*-1) and 1257bp (*exo70h4*-2) respectively in the single exon of *EXO70H4* gene were identified. The *vpy-2* (*Tnt1* insertion; line NF6898) mutant and *lin-4* mutant[4,5]. All mutants used in this study are derived from the R108 background. Error bars represent standard error of the mean. MC, microcolony. eIT, elongating infection thread in the root hairs. IT, fully elongated infection thread in root hairs. rIT, ramified infection thread into the cortex. NP, nodule primordia. N, nodule. 2-tailed Student's *t*-test, *, p<0.05; **, p<0.01; ***, p<0.001; ****, p<0.0001. Source data are provided as a Source Data file.

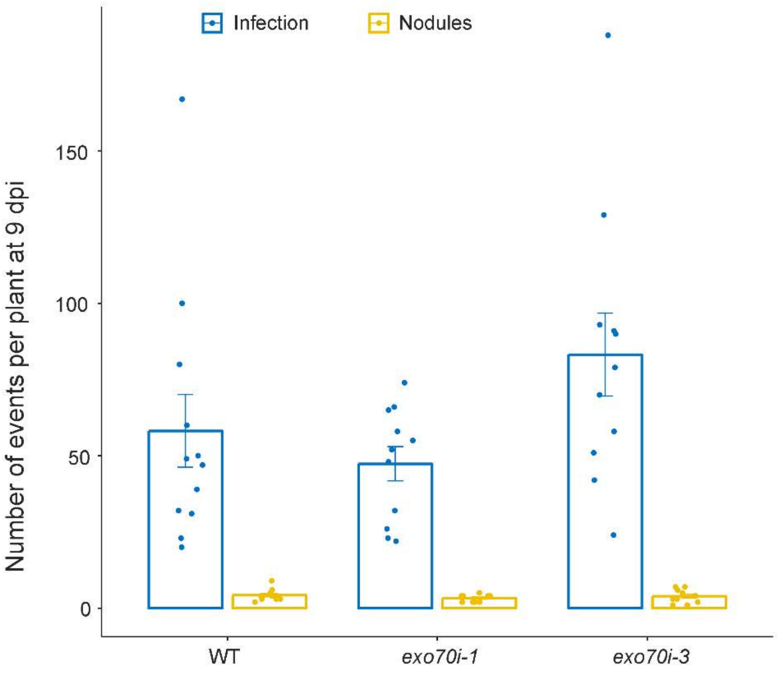

Supplementary Figure 19 Phenotypes of *exo70i* mutants in rhizobial infection. Quantification of infection events and nodules (and primordia) in WT and *exo70i* mutants at 9 dpi with *S. meliloti*. Error bars represent standard error of the mean. Source data are provided as a Source Data file.

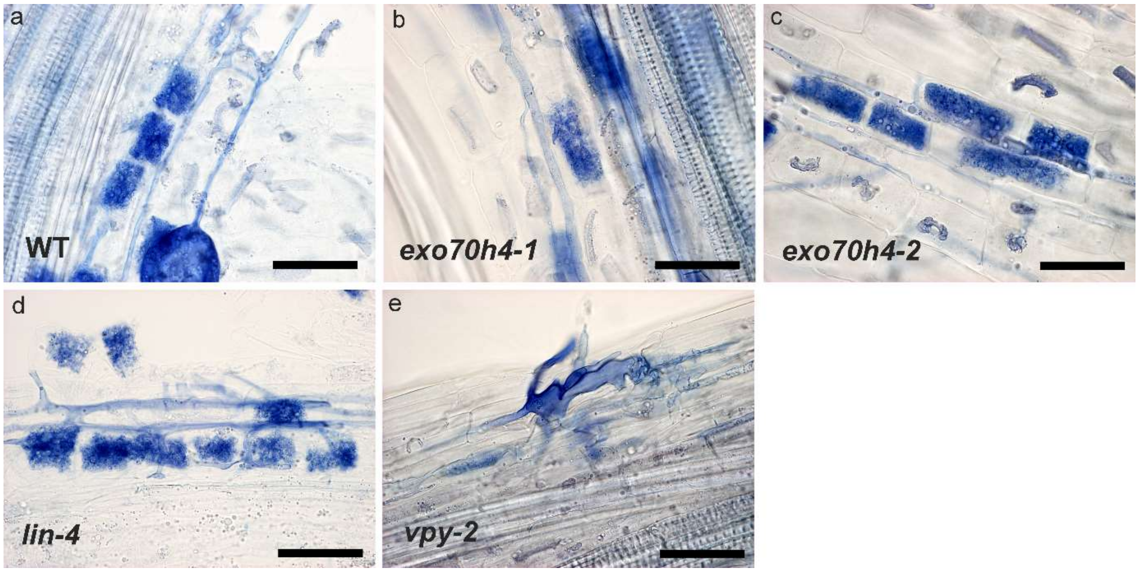

Supplementary Figure 20 Arbuscules are normal in *exo70h4* mutants. (a-e) Images of *M. truncatula* root cells 21 dpi with arbuscular mycorrhizal fungi *Rhizophagus irregularis (*syn*. Glomus intraradices)* showing normal arbuscules in all genotypes including both *exo70h4* alleles (**a-d**). In contrast, fungal entry within root tissues is compromisd in the *vpy-2* mutant (**e**). Blue colour indicates ink-stained arbuscules, hyphae or vesicles of arbuscular mycorrhizal fungi. Scale bars, 50 µm.

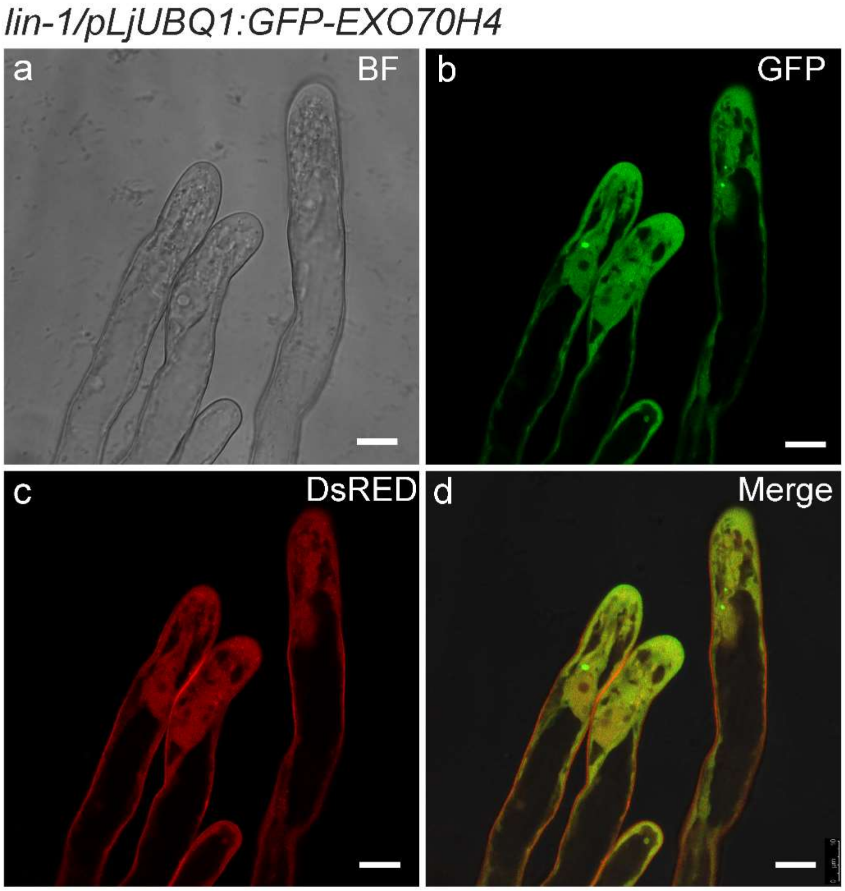

Supplementary Figure 21 Subcellular localization of EXO70H4 in *lin-1* after rhizobial inoculation. Confocal images from composite transgenic plants of GFP-EXO70H4 driven by *LjUBQ1* promoter showing subcellular localization of GFP-EXO70H4 in root hairs of *lin-1* mutant 7 dpi with *S. meliloti* Rm1021-CFP. DsRED was used as a transgenic marker. **a-d** Bright field, GFP, DsRED and merged images. DsRED and GFP were pseudo-coloured in red and green respectively. Scale bars, 10 µm.

Supplementary Table 1

**Sequencing primers**	
T7 sequencing primer F	5'-TAATACGACTCACTATAGGGC-3'
3'BD sequencing primer	5'-TTTTCGTTTTAAAACCTAAGAGTC-3'
3'AD sequencing primer	5'-AGATGGTGCACGATGCACAG-3'
**Cloning primers**	
MSP GW F	5'-CACCATGGATAGACTCATAAAGTTAG-3'
MSP GW R	5'-TGAGTCTTTTGAGTTAACTGATTC-3'
Ankyrin GW F	5'-CACCCAAGGTCAAACTTTGCTTCA-3'
Ankyrin GW R	5'-CTAAAGAACAGCCAAAGGCATTG-3'
VPY-LIKE_F	5'-ggggacaagtttgtacaaaaaagcaggctTCATGGATAGGTTGGTGAAAAC-3'
VPY-LIKE_R	5'-ggggaccactttgtacaagaaagctgggtTTCAAGCAAATGTCTCAGATT-3'
LIN_F	5'-ggggacaagtttgtacaaaaaagcaggctTCATGTCGGGGAATTTCAGATTC-3'
LIN_R	5'-ggggaccactttgtacaagaaagctgggtTTTAGTCCAATGCCCAGACCTGG-3'
proVPY_F	5'-CACTTACGCCGTGTTTGAATTG-3'
proVPY-R	5'-TTGGTTTAGGGTTTGTATTGAAAA-3'
proLIN_F	5'-ggggacaagtttgtacaaaaaagcaggctTCgatattaccatagcactgcac-3'
proLIN_R	5'-ggggaccactttgtacaagaaagctgggtTcaaattcatccgacataaagg-3'
LIN_1546bp-F	5'-ggggacaagtttgtacaaaaaagcaggctTCGATTTTGTTTGTCCAATCACAGG-3'
LIN_1948bp-F	5'-ggggacaagtttgtacaaaaaagcaggctTCGTGTTATCGCAAGCTGCAGTTG-3'
LIN_3588bp-F	5'-ggggacaagtttgtacaaaaaagcaggctTCAGTTGTAAAGAAGTAGTTGAG-3'
LIN_1546bp-R	5'-ggggaccactttgtacaagaaagctgggtTCTATTTTGGTGGTTTTGATCC-3'
LIN_3588bp-R	5'-ggggaccactttgtacaagaaagctgggtTCTAccacaactctgtcacatcaac-3'
LIN-like F	5'-ggggacaagtttgtacaaaaaagcaggctTCATGAACACAACACGAACTCAAATCC-3'
LIN-like R	5'-ggggaccactttgtacaagaaagctgggtTTTAATTCATTTCCCAAGCCTG-3'
**Genotyping primers**	
Tnt1 F	5'-TCCTTGTTGGATTGGTAGCC-3'
Tnt1 R	5'-CAGTGAACGAGCAGAACCTGTG-3'
vpy 6898 F	5'-CCAGGTGCAGCTATCAAAGA-3'
vpy 6898 R	5'-TGCAGCCCTATGAAGTGATG-3'
exo70h genotyping F	5'-CTAACCGTTCCTCCACCGATC-3'
exo70h genotyping R	5'-AACTTGCTCCACCGTCTTCT-3'

Supplementary References

1. Dereeper, A. et al. Phylogeny.fr: robust phylogenetic analysis for the non-specialist. *Nucleic Acids Res.* **36,** W465-W469 (2008).
2. Ciccarelli, F.D., Doerks,T., von Mering, C., Creevey, C.J., Snel, B., Bork, P. Toward automatic reconstruction of a highly resolved tree of life. *Science* **311,** 1283–87 (2006).
3. Breakspear, A., et al. The root hair "infectome" of *Medicago truncatula* uncovers changes in cell cycle genes and reveals a requirement for Auxin signaling in rhizobial infection. *Plant Cell* **26,** 4680–4701 (2014).
4. Guan, D. et al. Rhizobial infection is associated with the development of peripheral vasculature in nodules of *Medicago truncatula*. *Plant Physiol.* **162,** 107-115 (2013).
5. Murray, J.D. et al. Vapyrin, a gene essential for intracellular progression of arbuscular mycorrhizal symbiosis, is also essential for infection by rhizobia in the nodule symbiosis of *Medicago truncatula*. *Plant J.* **65,** 244–252 (2011).
