# Supplementary material for: A protein complex required for polar growth of rhizobial infection threads
Source: Nat Commun. 2019 Jun 28;10:2848. doi: 10.1038/s41467-019-10029-y (PMC6599036; doi:10.1038/s41467-019-10029-y)
Supplement: Supplementary file 2 — Description of Additional Supplementary Files [file 41467_2019_10029_MOESM2_ESM.pdf]

Description of Additional Supplementary Files

File Name: Supplementary Movie 1

Description: Dynamics of VPY-GFP labeled puncta in Medicago truncatula growing root hairs 3dpi with Sinorhizobium meliloti. The typical movement of nucleus-associated, VPY-labeled dots is shown in uninfected root hairs in an inoculated M. truncatula root. The movie is 108x faster than real time (41 images were acquired over 9min). Each image is a z-projection of a stack encompassing the 3 root hairs. DsRed fluorescence (pseudo-colored magenta) is merged to GFP fluorescence (pseudo-colored green). The imaged field is 100x125 µm.

File Name: Supplementary Movie 2

Description: Dynamics of VPY-GFP labeled puncta in a Medicago truncatula root hair undergoing infection by CFP-labeled Sinorhizobium meliloti. The movement of nucleus-associated and infection thread tip-associated VPY-labeled dots is shown in a M. truncatula root hair hosting an elongating infection thread. The movie is accelerated 150x compared to real time (39 images were acquired over 10 min). Each image is a z-projection of a stack encompassing the infection thread. CFP fluorescence of the rhizobia (pseudo-colored magenta) is merged to GFP fluorescence (pseudo-colored green). The imaged field is 60x60 µm.
